# Interplay of Orbital Overlap and Exciton Coupling in the Optical Properties of Adamantylethynyl‐Substituted Pyrene Molecular Crystals

**DOI:** 10.1002/chem.70949

**Published:** 2026-03-26

**Authors:** Benedikt Herbert, Kazutaka Shoyama

**Affiliations:** ^1^ Center for Nanosystems Chemistry (CNC) and Institut für Organische Chemie Universität Würzburg Würzburg Germany

**Keywords:** band structure, crystal growth, exciton coupling, PAH, photophysics

## Abstract

Molecular crystals of apolar π‐conjugated hydrocarbons exhibit pronounced solution‐to‐crystal spectral shifts, yet the interplay between band formation and exciton coupling remains unclear. Adamantylethynyl‐pyrene derivatives were designed to adopt unidirectional crystal packing, providing a model system to disentangle these effects. Large redshifts of up to −203 meV are observed, which cannot be explained by exciton coupling alone but require a substantial contribution from band dispersion, identified here as a narrowing of frontier crystal orbital energy levels. The remaining discrepancies between experiment and the full‐coherence limit correspond to coherence lengths of four to seven molecules, consistent with literature values. These results provide a basis for interpreting more complex organic crystalline materials and highlight the need for a more comprehensive understanding of the photophysical properties of molecular crystals.

## Graphical abstract

Sterically shielded adamantylethynyl‐substituted pyrenes form quasi‐one‐dimensional π‐stacks that enable direct quantification of solution‐to‐crystal spectral shifts. The optical response is dominated by band dispersion arising from frontier crystal orbitals, while excitonic contributions are secondary. This work establishes frontier crystal orbitals as a key concept for understanding the photophysics of apolar polycyclic aromatic hydrocarbon (PAH) crystals.

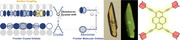

## 1 Introduction

**FIGURE 1:**
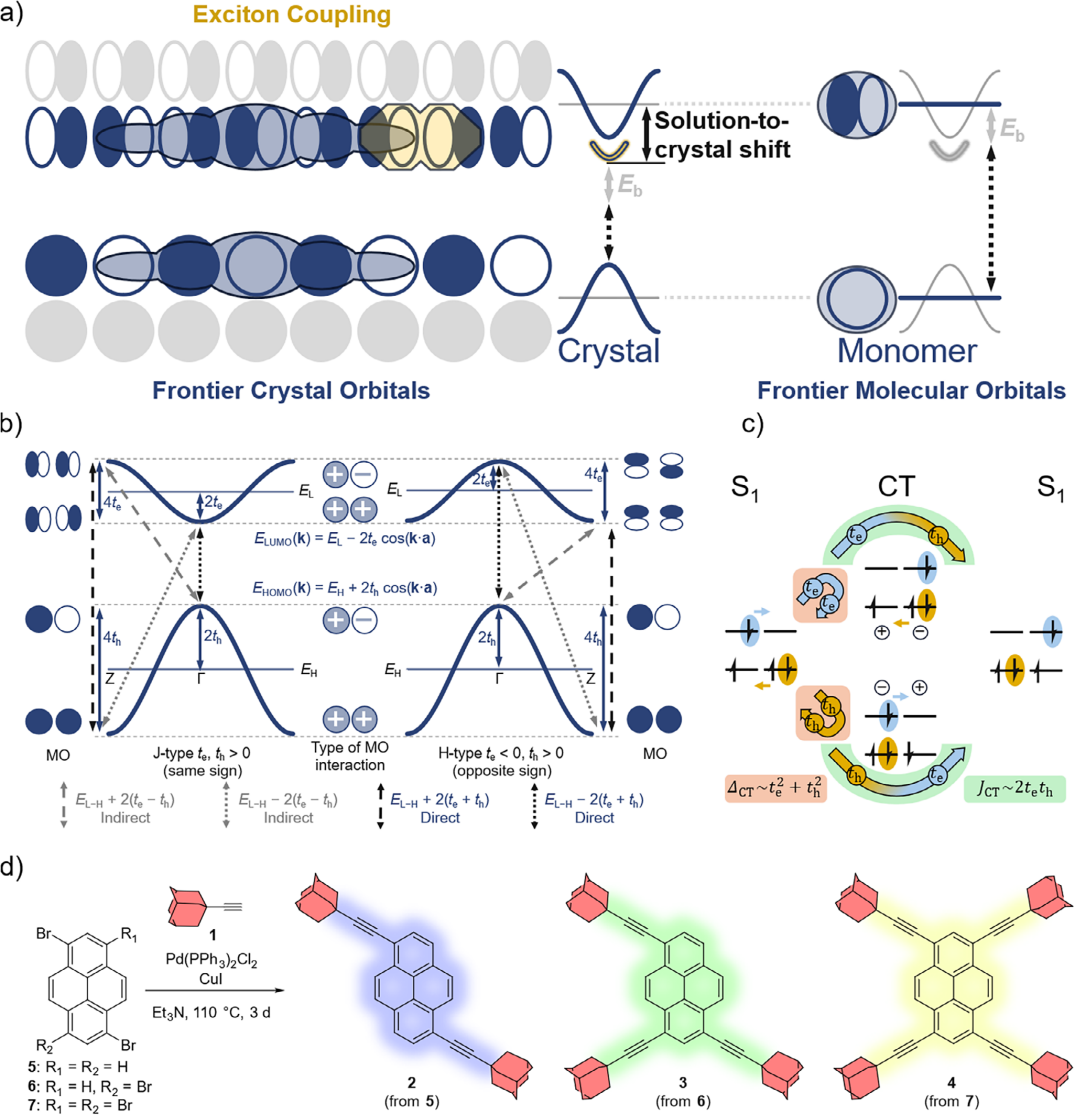
Schematic illustration of solution‐to‐crystal shift, related theory frameworks, and synthetic scheme for pyrene derivatives **2–4**. (a) Band structure and exciton coupling in crystal and monomer frontier crystal orbitals delocalized over approximately five molecules are illustrated as blue clouds in the crystal, whereas orbitals are localized on a single molecule in the monomer. CT‐mediated exciton coupling is indicated by an orange cloud. *E*_b_: exciton binding energy. (b) Band theory schemes of J‐type (left) and H‐type (right) based on charge transfer integrals *t*_e_ and *t*_h_. (c) Schematic illustration of CT‐mediated exciton coupling. (d) Synthesis of pyrenes **2–4** from ethynyladamantane **1**. The background colors are the respective apparent emission colors of crystals.

Crystals of apolar polycyclic aromatic hydrocarbons (PAHs) exhibit rich solid‐state optical properties arising from the interplay of intermolecular orbital interactions and excitonic effects governed by London‐dispersion‐controlled packing geometry (Figure 1a) [1, 2, 3, 4, 5, 6, 7, 8]. These features manifest as solution‐to‐crystal shifts commonly observed when comparing the colors of crystals with those of isolated molecules in solution. Despite their fundamental importance, scientific analyses of such shifts have often been overshadowed by the rapid growth of applications in organic semiconductors and luminescent materials. Two theoretical frameworks are typically used, yet they are rarely considered together. Band‐structure approaches [2, 7, 9, 10, 11, 12, 13] attribute the shift to stabilization and destabilization of frontier orbitals through short‐range intermolecular overlap, producing dispersion of valence and conduction bands when delocalization is significant (Figure 1b). Exciton models [5, 6, 14, 15, 16, 17] instead treat the excited state as a manifold of localized Frenkel excitations that interact through both classical long‐range Coulombic coupling [5, 16] and short‐range charge‐transfer (CT) mixing [6, 15, 17] (Figure 1c). This excitonic framework explains exciton splitting, H/J behavior, and vibronic progression, but generally holds the monomer excitation energy fixed and therefore cannot capture orbital‐interaction‐driven energy shifts. Although actual PAH crystals often fall between these limits, their multidirectional packing interactions hinder a straightforward assignment to either viewpoint. Systems in which interactions are dominated along a single crystallographic direction offer a route to disentangle the relative magnitudes of band effects and exciton coupling.

Here, we show that short‐range orbital interactions, giving rise to delocalized frontier crystal orbitals, dominate the experimentally observed solution‐to‐crystal shifts of a family of apolar PAHs, adamantylethynyl‐substituted pyrenes (Figure 1a,d). Their steric design enforces unidimensional π‐stacking while suppressing interactions in other directions. Analysis of frontier orbital energies derived from representative dimers shows that band‐structure effects account for most of the observed redshift, whereas exciton coupling contributions are an order of magnitude smaller. These findings indicate that for densely packed, apolar PAHs with strong local π‐overlap and weak Coulombic screening, band structure predominantly governs the solid‐state optical properties, with excitonic interactions providing a secondary contribution. This insight lays the groundwork for a more comprehensive understanding of the optical behavior of organic molecular crystals and underscores the need for frameworks that integrate both band‐like and excitonic perspectives.

## 2 Results and Discussion

A series of adamantylethynyl‐substituted pyrenes **2–4** (Figure 1) was designed to enforce unidimensional π–π interactions while suppressing multidirectional contacts through steric shielding by the adamantane groups. Sonogashira coupling of di‐, tri‐, and tetra‐bromopyrenes (**5–7**) [18, 19, 20, 21, 22] with (1‐adamantyl)acetylene **1** [23] afforded pyrenes **2–4**, characterized by high‐resolution mass spectrometry (HRMS), ^1^H and ^13^C NMR, UV/Vis, and fluorescence spectroscopy (see Supporting Information).

**FIGURE 2:**
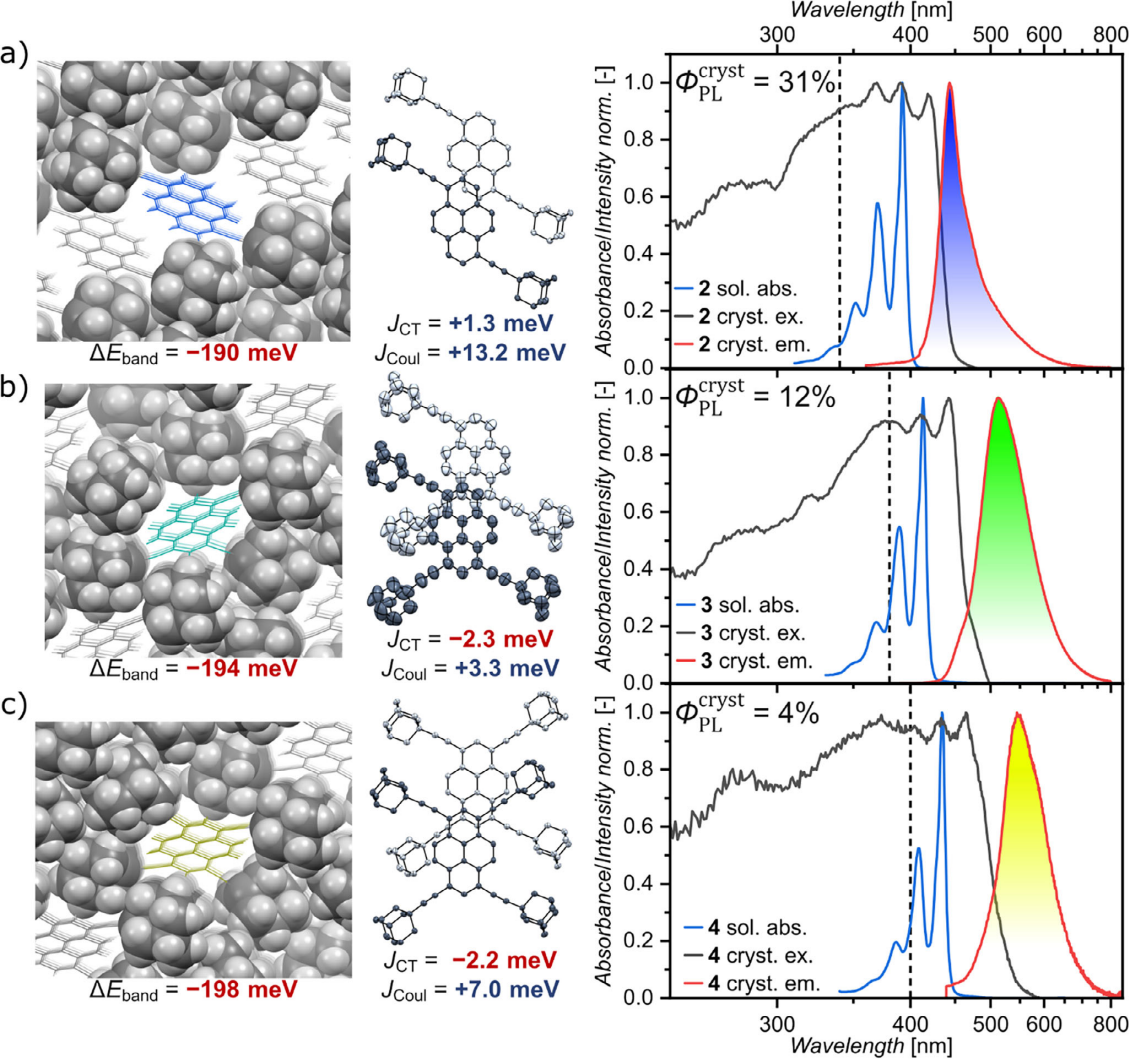
Crystal structure and photophysical properties of adamantylethynyl‐substituted pyrenes (a) **2**, (b) **3**, and (c) **4**. Left: Crystal structure along the π‐stacking direction with Δ*E*_band_ (vide infra). Middle: π‐Stacking dimer with exciton couplings (vide infra). Ellipsoids drawn at 50% probability. Right: Blue line; normalized absorption spectrum in CH_2_Cl_2_ solution at 295 K, gray line; normalized excitation spectrum of crystallite ensemble at 295 K, red line; normalized emission spectrum of crystallite ensemble at 295 K. Area under red line: apparent emission color (Figures S29–S31). The dashed vertical line marks the excitation wavelength for emission spectroscopy at (a) 340, (b) 380, and (c) 400 nm. The corresponding absolute photoluminescence quantum yields of crystalline solids can be found inside the respective graphs.

Single crystals of **2–4** suitable for X‐ray crystallographic analyses were obtained from their CH_2_Cl_2_ solution by slow diffusion of antisolvent, either MeOH or *n*‐C_6_H_14_, and all exhibit the intended quasi‐one‐dimensional π‐stacking motif (Figures 2, S32–S34) [24]. In all three crystal structures, the adamantyl groups suppress inter‐stack contacts and enforce π‐overlap exclusively along a single stacking direction. In di‐substituted pyrene **2**, channels of π‐stacked pyrene cores are formed that are laterally isolated by surrounding adamantyl groups. Tri‐substituted pyrene **3** displays an even higher degree of stack isolation, with each π‐stack separated by adamantyl groups in all directions. Tetra‐substituted pyrene **4** similarly adopts completely isolated one‐dimensional π‐stacks. The resulting variations in slip distance and overlap area directly modulate the intermolecular orbital interactions and thereby the solid‐state photophysical behavior of **2–4** (Figure 2).

We assessed the solution‐to‐crystal shifts of **2–4** by comparing the UV/Vis absorption spectra in CH_2_Cl_2_ with the excitation spectra of ensembles of crystals (Figure 2). The ensemble measurements represent randomly oriented crystallites; anisotropic spectral features are therefore averaged out. The absorption maxima of **2–4** in solution appear at 392, 413, and 436 nm, respectively, whereas the excitation maxima of the crystalline ensembles are observed at 419, 442, and 464 nm (Table S1). These correspond to solution‐to‐crystal shifts (Δ*E*_obs_) of −203, −196, and −184 meV for **2–4**, respectively.

We estimated the contribution of direct intermolecular orbital interactions to the spectral shifts by assuming that the destabilization of the highest occupied and stabilization of the lowest unoccupied orbitals of the crystal, that is, frontier crystal orbitals (Figure 1a), results in a corresponding narrowing of the first electronic transition of the crystal relative to the isolated monomer in solution. Within a one‐dimensional tight‐binding framework, twice the dimer–monomer HOMO–LUMO energy gap narrowing approximates that of the frontier crystal orbitals (Δ*E*_band_). The dimer and monomer frontier orbital energy levels were calculated at the B3LYP/def2svp level of theory. We intentionally employed the B3LYP functional, despite its known tendency to over‐delocalize orbitals in weakly interacting systems [25], because this behavior offers an upper‐bound estimate for the degree of orbital delocalization expected in the frontier crystal orbitals in the band structure. Using this approach, the estimated band‐structure contributions to the spectral shifts, Δ*E*_band_, are −190, −194, and −198 meV for **2–4**, respectively (Table 1). An alternative estimation based on electron and hole CT integrals (*t*_e_ and *t*_h_) between isolated monomers as −2(|*t*_e_| + |*t*_h_|) predict 22–47 meV smaller shifts than the dimer‐based values, because the dimer frontier orbital calculations capture additional energy reorganization induced by orbital deformation [26, 27] through π–π contact (Tables 1 and S5).

**TABLE 1:** Calculated transfer integrals and spectral shifts of **2–4**. All energies in meV^a^.

Compound	Δ*E*_obs_	*t*_e_	*t*_h_	Δ*E*_band_	*Δ*_CT_	*J*_CT_	*J*_Coul_	Δ*E*_exciton_	Δ*E*_total_
**2**	−203	+19.0	−65.0	−190	−4.8	+1.3	+13.2	−33.8	−224
**3**	−196	+18.5	+59.0	−194	−8.0	−2.3	+3.3	−19.2	−213
**4**	−184	+26.1	+49.3	−198	−5.2	−2.2	+7.0	−23.6	−222

^a^ Δ*E*_obs_: Observed solution‐to‐crystal shift using absorption spectra in CH_2_Cl_2_ and crystal excitation spectra. *t*_e_, *t*_h_: Electron and hole transfer integrals between neighboring molecules calculated at the PW91/TZP level of theory. Signs are assigned so that *t*_h_ > 0 [6]. Δ*E*_band_: Calculated band dispersion width according to −2(Δ*E*_HOMO_ + Δ*E*_LUMO_), where Δ*E*_HOMO_ and Δ*E*_LUMO_ are the energy difference between monomer and dimer frontier orbitals calculated at the B3LYP/def2svp level of theory. This contains band dispersion width from the transfer integral defined as 2(|*t*_e_| + |*t*_h_|) and stabilization through orbital deformation upon dimer formation. *Δ*_CT_, *J*_CT_: Site energy and CT‐mediated exciton coupling calculated as *J*_CT_ = −2*t*_e_ *t*_h_/(*E*_CT_ − *E*_S1_), *Δ*_CT_ = −2(*t*_e_^2^ + *t*_h_^2^)/(*E*_CT_ − *E*_S1_), where *E*_CT_ is calculated as the energy difference between ground state dimer M^0^M^0^ and charge separated dimer M^+^M^−^ by cDFT at the ωB97XD/def2svp level of theory [6]. *E*_S1_ calculated by monomer TDDFT at the B3LYP/def2svp level of theory, which effectively reproduced the experimental absorption maximum in CH_2_Cl_2_. *J*_Coul_: Long‐range Coulombic exciton coupling calculated by the TrESP method at the B3LYP/def2svp level of theory. Δ*E*_exciton_: Upper redshift limit of Coulombic and CT‐mediated exciton coupling defined as *Δ*_CT_ − 2|*J*_CT_| − 2|*J*_Coul_|. Δ*E*_total_: Estimated upper limit of solution‐to‐crystal shift by accounting band‐like frontier crystal orbital formation and exciton coupling defined as Δ*E*_band_ + Δ*E*_exciton_. Details in Supporting Information.

For **2**, the crystal's lowest electronic transition is indirect, that is forbidden, as indicated by the nodal pattern of the molecular orbitals (Figure S35) and the opposite signs of the electron and hole transfer integrals (Table 1, here relative signs are assigned according to Spano et al. [6]). This formally predicts much smaller orbital‐energy narrowing for the direct, that is allowed, transition, and a blueshift for CT‐mediated exciton coupling (Figure 1b, vide infra). In this study, we use the predicted indirect transition of **2** as the allowed budget for attributing the observed spectral redshift. Overall, the band‐structure‐driven orbital destabilization/stabilization of the frontier crystal orbitals accounts for most of the experimentally observed solution‐to‐crystal shift, but not entirely, indicating that additional factors, such as exciton coupling, must also contribute.

As secondary contributions, we evaluated exciton coupling arising from both Coulombic and CT‐mediated mechanisms (Table 1). As expected for apolar chemical structure, the classical Coulombic exciton coupling (*J*_Coul_) computed using the transition‐charge‐from‐electrostatic‐potential (TrESP) method [28] reaches at most +13.2 meV for compound **2**. Exciton coupling through the CT‐state was estimated by applying second‐order perturbation theory, based on CT integrals (details in Supporting Information) [6]. The computed CT‐mediated exciton coupling (*J*_CT_) was only +1.3, −2.3, −2.2 meV for **2–4**, respectively. The site‐energy correction arising from coupling with CT‐state (*Δ*_CT_, Figure 1c) has also been estimated with −4.8, −8.0, and −5.2 meV for **2–4**, respectively. The overall excitonic budget (Δ*E*_exciton_) for the solution‐to‐crystal red shift was estimated under 1D tight‐binding conditions as *Δ*_CT_ − 2|*J*_CT_| − 2|*J*_Coul_|, which resulted in −33.8, −19.2, and −23.6 meV for **2–4**, respectively. These values account only up to approximately 15% of the maximum shift estimated by band dispersion.

**FIGURE 3:**
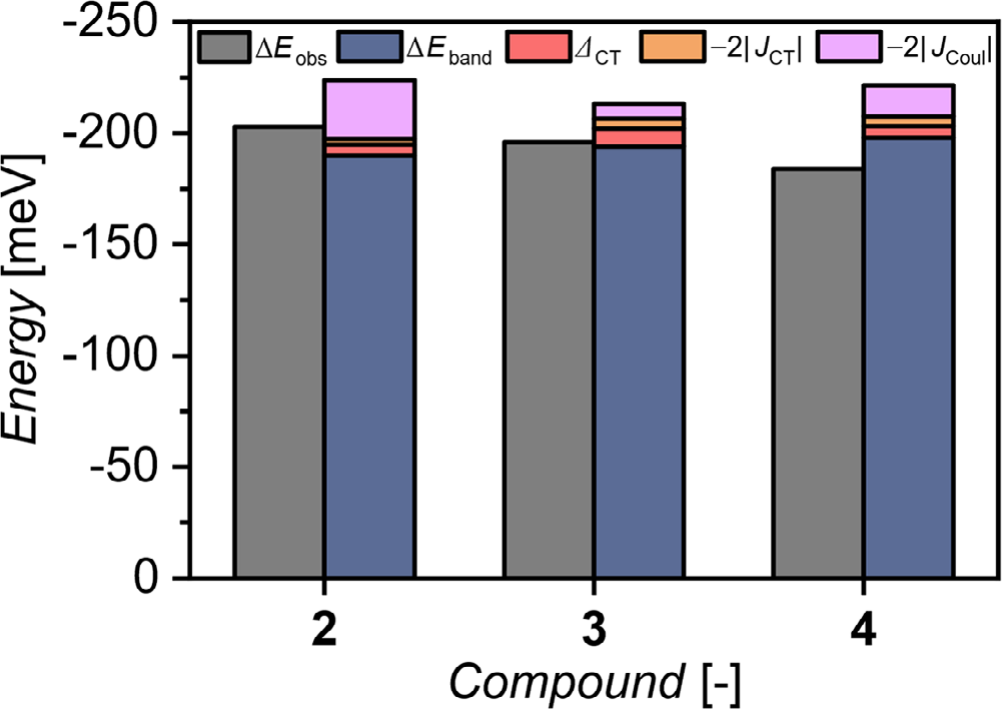
Comparison of observed solution‐to‐crystal shift (Δ*E*_obs_) and the sum of estimated budget, that is, maximum redshift under hypothetical perfect coherence, of redshift originating from band structure (Δ*E*_band_), site energy correction (*Δ*_CT_), CT‐mediated exciton coupling (−2|*J*_CT_|), and Coulombic exciton coupling (−2|*J*_Coul_|).

The overall budget for the solution‐to‐crystal shift (Δ*E*_total_) was estimated as the sum of band‐structure and exciton‐coupling contributions, evaluated as Δ*E*_band_ + Δ*E*_exciton_. This value (Δ*E*_total_) represents the spectral shift expected in the hypothetical limit where perfectly coherent band contributions (Δ*E*_band_) and localized excitonic contributions (Δ*E*_exciton_) coexist. The value for the real system (Δ*E*_obs_) can subsequently be described as an intermediate case by introducing an overall coherence length (vide infra). The negative signs reflect that we consistently use the lowest‐energy excitonic or band‐edge transition for comparison with experiment, even when this state is formally H‐type (Coulombic or CT‐mediated) or indirect, but becomes weakly allowed in real crystals. The resulting Δ*E*_total_ values for **2–4** are −224, −213, and −222 meV (Table 1). These values effectively account for the majority (ca. 90%) of experimental shift (Δ*E*_obs_) (Figure 3). One reason for the slightly smaller Δ*E*_obs_ than Δ*E*_total_ can be the nonideal coherence of the system, as perfect coherence is assumed under tight‐binding conditions. Because the band‐structure and excitonic contributions originate from different electronic states and opposite degrees of (de)localization—the former assuming delocalized ground‐state frontier orbitals, the latter localized excited states—their individual coherence lengths cannot be straightforwardly estimated. Nevertheless, the upper limits for the apparent combined coherence length (*N*_coh_) were estimated using the relation Δ*E*_obs_ = Δ*E*_total_ cos(π/(*N*_coh_+1)), which is derived from the solution of a one‐dimensional tight‐binding chain containing *N*_coh_ coherently interacting units [6]. This analysis gives values 6.3, 6.9, and 4.3 molecules for **2–4**, respectively, which match the range of reported band delocalization lengths [29] and exciton coherence [6, 30].

We here note that other potential contributions, such as changes in molecular geometry and dielectric effects, are considered nondominant. The PAHs studied here are rigid, and thus, negligible geometrical reorganization is expected. Dielectric effects are also weak for these apolar PAHs (Table S7). Thus, the majority of the solution‐to‐crystal shift is attributed to band and excitonic effects.

## 3 Conclusion

In summary, we have shown that the solution‐to‐crystal spectral shift for apolar adamantylethynyl‐pyrene derivatives **2–4** can be accounted for by band dispersion and exciton coupling, with band dispersion constituting the dominant contribution and excitonic contributions being an order of magnitude smaller. The adamantylethynyl substitution provides steric shielding, leading to quasi‐one‐dimensional stacks that confine solid‐state effects to a quantifiable framework. The predominance of band dispersion, that is, frontier crystal orbital energy shifts, over excitonic contributions in attributing the experimental spectral shift highlights the importance of a molecular‐orbital‐from‐atomic‐orbital perspective for understanding the crystal‐state properties of apolar PAHs. At the same time, the necessity of including excitonic contributions to fully explain the crystal spectral features underscores the complexity of organic molecular crystals and emphasizes the need for a more general framework that integrates both band and excitonic descriptions. We are currently extending this approach to more complex doped molecular crystals, including crystalline solid solutions [31, 32, 33], where controlled perturbations of crystal packing and orbital interactions are expected to provide additional handles for tailoring solid‐state optical properties.

## Conflicts of Interest

The authors declare no conflicts of interest.

## Supporting information



The authors have cited additional references within the Supporting Information [34, 35, 36, 37, 38, 39, 40, 41, 42, 43, 44, 45, 46, 47].

## References

[1] J. Gierschner , J. Shi , B. Milián‐Medina , D. Roca‐Sanjuán , S. Varghese , and S. Park , “Luminescence in Crystalline Organic Materials: From Molecules to Molecular Solids,” Advanced Optical Materials 9 (2021): 2002251, 10.1002/adom.202002251.

[2] J.‐L. Brédas , D. Beljonne , V. Coropceanu , and J. Cornil , “Charge‐Transfer and Energy‐Transfer Processes in π‐Conjugated Oligomers and Polymers: A Molecular Picture,” Chemical Reviews 104 (2004): 4971–5004. 15535639 10.1021/cr040084k

[3] R. Ramakrishnan , M. A. Niyas , M. P. Lijina , and M. Hariharan , “Distinct Crystalline Aromatic Structural Motifs: Identification, Classification, and Implications,” Accounts of Chemical Research 52 (2019): 3075–3086, 10.1021/acs.accounts.9b00320. 31449389

[4] P. Naumov , S. Chizhik , M. K. Panda , N. K. Nath , and E. Boldyreva , “Mechanically Responsive Molecular Crystals,” Chemical Reviews 115 (2015): 12440–12490, 10.1021/acs.chemrev.5b00398. 26535606

[5] F. Würthner , T. E. Kaiser , and C. R. Saha‐Möller , “J‐Aggregates: From Serendipitous Discovery to Supramolecular Engineering of Functional Dye Materials,” Angewandte Chemie International Edition 50 (2011): 3376–3410, 10.1002/anie.201002307. 21442690

[6] N. J. Hestand and F. C. Spano , “Expanded Theory of H‐ and J‐Molecular Aggregates: The Effects of Vibronic Coupling and Intermolecular Charge Transfer,” Chemical Reviews 118 (2018): 7069–7163, 10.1021/acs.chemrev.7b00581. 29664617

[7] R. Hoffmann , “How Chemistry and Physics Meet in the Solid State,” Angewandte Chemie International Edition in English 26 (1987): 846–878, 10.1002/anie.198708461.

[8] J. P. Wagner and P. R. Schreiner , “London Dispersion in Molecular Chemistry—Reconsidering Steric Effects,” Angewandte Chemie International Edition 54 (2015): 12274–12296, 10.1002/anie.201503476. 26262562

[9] R. L. Carey , X. Ren , I. E. Jacobs , et al., “Long Spin Lifetimes of Charge Carriers in Rubrene Crystals Due to Fast Transient‐Localization Motion,” Nature Communications 16 (2025): 7605, 10.1038/s41467-025-62830-7. PMC12356851 40817376

[10] P. M. Kazmaier and R. Hoffmann , “A Theoretical Study of Crystallochromy. Quantum Interference Effects in the Spectra of Perylene Pigments,” Journal of the American Chemical Society 116 (1994): 9684–9691, 10.1021/ja00100a038.

[11] M. C. R. Delgado , K. R. Pigg , D. A. da Silva Filho , et al., “Impact of Perfluorination on the Charge‐Transport Parameters of Oligoacene Crystals,” Journal of the American Chemical Society 131 (2009): 1502–1512, 10.1021/ja807528w. 19173667

[12] F. Provencher , N. Bérubé , J.‐F. Laprade , et al., “Large Electronic Bandwidth in Solution‐Processable Pyrene Crystals: The Role of Close‐Packed Crystal Structure,” Journal of Chemical Physics 137 (2012): 034706, 10.1063/1.4732504. 22830723

[13] M. Schwarze , W. Tress , B. Beyer , et al., “Band Structure Engineering in Organic Semiconductors,” Science 352 (2016): 1446–1449, 10.1126/science.aaf0590. 27313043

[14] N. J. Hestand , H. Yamagata , B. Xu , et al., “Polarized Absorption in Crystalline Pentacene: Theory vs Experiment,” Journal of Physical Chemistry C 119 (2015): 22137–22147, 10.1021/acs.jpcc.5b07163.

[15] L. Gisslén and R. Scholz , “Crystallochromy of Perylene Pigments: Interference Between Frenkel Excitons and Charge‐Transfer States,” Physical Review B 80 (2009): 115309.

[16] M. Kasha , H. R. Rawls , and M. Ashraf El‐Bayoumi , “The Exciton Model in Molecular Spectroscopy,” Pure and Applied Chemistry 11 (1965): 371–392, 10.1351/pac196511030371.

[17] N. J. Hestand , R. V. Kazantsev , A. S. Weingarten , L. C. Palmer , S. I. Stupp , and F. C. Spano , “Extended‐Charge‐Transfer Excitons in Crystalline Supramolecular Photocatalytic Scaffolds,” Journal of the American Chemical Society 138 (2016): 11762–11774, 10.1021/jacs.6b05673. 27589150

[18] T. Iwasaki , S. Murakami , Y. Takeda , et al., “Molecular Packing and Solid‐State Photophysical Properties of 1,3,6,8‐Tetraalkylpyrenes,” Chemistry—A European Journal 25 (2019): 14817–14825, 10.1002/chem.201903224. 31410873

[19] F. Xu , T. Nishida , K. Shinohara , et al., “Trimethylsilyl Group Assisted Stimuli Response: Self‐Assembly of 1,3,6,8‐Tetrakis((trimethysilyl)Ethynyl)Pyrene,” Organometallics 36 (2017): 556–563, 10.1021/acs.organomet.6b00781.

[20] S. Bernhardt , M. Kastler , V. Enkelmann , M. Baumgarten , and K. Müllen , “Pyrene as Chromophore and Electrophore: Encapsulation in a Rigid Polyphenylene Shell,” Chemistry—A European Journal 12 (2006): 6117–6128, 10.1002/chem.200500999. 16847838

[21] H. Maeda , T. Maeda , K. Mizuno , K. Fujimoto , H. Shimizu , and M. Inouye , “Alkynylpyrenes as Improved Pyrene‐Based Biomolecular Probes With the Advantages of High Fluorescence Quantum Yields and Long Absorption/Emission Wavelengths,” Chemistry—A European Journal 12 (2006): 824–831, 10.1002/chem.200500638. 16267869

[22] Y. Sagara and T. Kato , “Stimuli‐Responsive Luminescent Liquid Crystals: Change of Photoluminescent Colors Triggered by a Shear‐Induced Phase Transition,” Angewandte Chemie International Edition 47 (2008): 5175–5178, 10.1002/anie.200800164. 18512835

[23] M. W. Lee , Y. V. Sevryugina , A. Khan , and S. Q. Ye , “Carboranes Increase the Potency of Small Molecule Inhibitors of Nicotinamide Phosphoribosyltranferase,” Journal of Medicinal Chemistry 55 (2012): 7290–7294, 10.1021/jm300740t. 22889195

[24] Deposition numbers 2518021 (for **2**), 2518022 (for **3**), and 2518023 (for **4**) contain the supplementary crystallographic data for this paper. These data are provided free of charge by the joint Cambridge Crystallographic Data Centre and Fachinformationszentrum Karlsruhe Access Structures service.

[25] A. J. Cohen , P. Mori‐Sánchez , and W. Yang , “Insights Into Current Limitations of Density Functional Theory,” Science 321 (2008): 792–794, 10.1126/science.1158722. 18687952

[26] S. Refaely‐Abramson , S. Sharifzadeh , M. Jain , R. Baer , J. B. Neaton , and L. Kronik , “Gap Renormalization of Molecular Crystals From Density‐Functional Theory,” Physical Review B 88 (2013): 081204, 10.1103/PhysRevB.88.081204.

[27] Y. Kang , S. H. Jeon , Y. Cho , and S. Han , “Ab Initio Calculation of Ionization Potential and Electron Affinity in Solid‐State Organic Semiconductors,” Physical Review B 93 (2016): 035131, 10.1103/PhysRevB.93.035131.

[28] M. E. Madjet , A. Abdurahman , and T. Renger , “Intermolecular Coulomb Couplings From Ab Initio Electrostatic Potentials: Application to Optical Transitions of Strongly Coupled Pigments in Photosynthetic Antennae and Reaction Centers,” The Journal of Physical Chemistry B 110 (2006): 17268–17281, 10.1021/jp0615398. 16928026

[29] T. Sakanoue and H. Sirringhaus , “Band‐Like Temperature Dependence of Mobility in a Solution‐Processed Organic Semiconductor,” Nature Materials 9 (2010): 736–740, 10.1038/nmat2825. 20729848

[30] S. Tanaka , K. Miyata , T. Sugimoto , et al., “Enhancement of the Exciton Coherence Size in Organic Semiconductor by Alkyl Chain Substitution,” The Journal of Physical Chemistry C 120 (2016): 7941–7948, 10.1021/acs.jpcc.5b12686.

[31] O. Bolton , K. Lee , H.‐J. Kim , K. Y. Lin , and J. Kim , “Activating Efficient Phosphorescence From Purely Organic Materials by Crystal Design,” Nature Chemistry 3 (2011): 205–210, 10.1038/nchem.984. 21336325

[32] F. Unger , D. Lepple , M. Asbach , et al., “Optical Absorption Properties in Pentacene/Tetracene Solid Solutions,” The Journal of Physical Chemistry A 128 (2024): 747–760, 10.1021/acs.jpca.3c06737. 38232326

[33] B. Herbert , J. Walpuski , M. Stolte , and K. Shoyama , “Designing Organic π‐Conjugated Molecules for Crystalline Solid Solutions: Adamantane‐Substituted Naphthalenes,” ChemPlusChem 89 (2024): e202300761, 10.1002/cplu.202300761. 38259048

[34] J. P. Schaefer , M. J. Dagani , and D. S. Weinberg , “Secondary Isotope Effects in the Solvolysis of Norbornyl Bromides,” Journal of the American Chemical Society 89 (1967): 6938–6944, 10.1021/ja01002a023.

[35] W. A. Chalifoux , M. J. Ferguson , R. McDonald , F. Melin , L. Echegoyen , and R. R. Tykwinski , “Adamantyl‐Endcapped Polyynes,” Journal of Physical Organic Chemistry 25 (2012): 69–76, 10.1002/poc.1874.

[36] K. Thakur , D. Wang , S. Mirzaei , and R. Rathore , “Electron‐Transfer‐Induced Self‐Assembly of a Molecular Tweezer Platform,” Chemistry—A European Journal 26 (2020): 14085–14089, 10.1002/chem.202001919. 32608146

[37] W. Kabsch , “XDS,” Acta Crystallographica Section D Biological Crystallography 66 (2010): 125–132, 10.1107/S0907444909047337. 20124692 PMC2815665

[38] G. M. Sheldrick , “SHELXT—Integrated Space‐Group and Crystal‐structure Determination,” Acta Crystallographica Section A Foundations and Advances 71 (2015): 3–8, 10.1107/S2053273314026370. 25537383 PMC4283466

[39] G. M. Sheldrick , “Crystal Structure Refinement With SHELXL,” Acta Crystallographica Section C Structural Chemistry 71 (2015): 3–8, 10.1107/S2053229614024218. 25567568 PMC4294323

[40] C. B. Hübschle , G. M. Sheldrick , and B. Dittrich , “ShelXle: A Qt Graphical User Interface for SHELXL,” Journal of Applied Crystallography 44 (2011): 1281–1284, 10.1107/S0021889811043202. 22477785 PMC3246833

[41] A. L. Spek , “checkCIF Validation ALERTS: What They Mean and How to Respond,” Acta Crystallographica Section E Crystallographic Communications 76 (2020): 1–11, 10.1107/S2056989019016244. 31921444 PMC6944088

[42] A. L. Spek , “Single‐Crystal Structure Validation With the Program PLATON,” Journal of Applied Crystallography 36 (2003): 7–13, 10.1107/S0021889802022112.

[43] M. J. Frisch , G. W. Trucks , H. B. Schlegel , et al., Gaussian 16, Revision A.03 (Gaussian, Inc., 2016).

[44] Y. Shao , Z. Gan , E. Epifanovsky , et al., “Advances in Molecular Quantum Chemistry Contained in the Q‐Chem 4 Program Package,” Molecular Physics 113 (2015): 184–215, 10.1080/00268976.2014.952696.

[45] E. J. Baerends , N. F. Aguirre , N. D. Austin , et al., “The Amsterdam Modeling Suite,” The Journal of Chemical Physics 162 (2025): 162501, 10.1063/5.0258496. 40260801

[46] R. Dennington , T. A. Keith , and J. M. Millam , GaussView, Version 6.0.16 (Semichem Inc., 2016).

[47] T. Lu and F. Chen , “Multiwfn: A Multifunctional Wavefunction Analyzer,” Journal of Computational Chemistry 33 (2012): 580–592, 10.1002/jcc.22885. 22162017

