# Supplementary material for: Interplay of Orbital Overlap and Exciton Coupling in the Optical Properties of Adamantylethynyl‐Substituted Pyrene Molecular Crystals
Source: Chemistry. 2026 Mar 26;32(22):e70949. doi: 10.1002/chem.70949 (PMC13250353; doi:10.1002/chem.70949)
Supplement: Supplementary file 1 — The authors have cited additional references within the Supporting Information [34, 35, 36, 37, 38, 39, 40, 41, 42, 43, 44, 45, 46, 47]. [file CHEM-32-e70949-s001.pdf]

Supporting Information

Interplay of Orbital Overlap and Exciton Coupling in the Optical Properties of Adamantylethynyl-Substituted Pyrene Molecular Crystals

Benedikt Herbert,[a] and Kazutaka Shoyama*[a]

[a] B. Herbert, Dr. K. Shoyama
Center for Nanosystems Chemistry (CNC) and Institut für Organische Chemie,
Universität Würzburg
Am Hubland, 97074 Würzburg, Germany
E-mail: kazutaka.shoyama@uni-wuerzburg.de

Abstract: Molecular crystals of apolar π-conjugated hydrocarbons exhibit pronounced solution-to-crystal spectral shifts, yet the interplay between band formation and exciton coupling remains unclear. Adamantylethynyl-pyrene derivatives were designed to adopt unidirectional crystal packing, providing a model system to disentangle these effects. Large red shifts of up to 203 meV are observed, which cannot be explained by exciton coupling alone but require a substantial contribution from band dispersion, identified here as a narrowing of frontier crystal orbital energy levels. The remaining discrepancies between experiment and the full-coherence limit correspond to coherence lengths of four to seven molecules, consistent with literature values. These results provide a basis for interpreting more complex organic crystalline materials and highlight the need for a more comprehensive understanding of the photophysical properties of molecular crystals.

Table of Contents

Experimental Procedures 3
General Procedures 3
Synthesis 4
Vinyl bromide (S1) 4
1-Ethynyladamantane (1) 4
1,3,6-Tribromopyrene (6) 4
1,6-Bis[(1-adamantyl)ethynyl]pyrene (2) 5
1,3,6-Tris[(1-adamantyl)ethynyl]pyrene (3) 5
1,3,6,8-Tetrakis[(1-adamantyl)ethynyl]pyrene (4) 6
NMR Spectra 7
Mass Spectra 11
Optical Spectroscopy 13
Crystallography 22
Density Functional Theory (DFT) Calculations 29
Author contributions 39
References 39

Experimental Procedures

General Procedures

Chemicals and solvents
Chemicals and solvents were purchased from commercial suppliers and used without further purification unless otherwise noted.

Column chromatography
Column chromatography was performed using standard glass columns of different sizes, packed with silica-gel (particle size: 40–63 µm) purchased from Macherey-Nagel as stationary phase.

Thin-layer chromatography
For monitoring of the reaction progress, thin layer chromatography (TLC) was carried out, using TLC plates ALUGRAM®Xtra SIL G/UV254 (layer thickness: 0.2 mm) purchased from Marcherey-Nagel.

Melting points
Melting points were measured on an optical microscope (Olympus BX41) with an applied heating rate of 5 K min^{-1}.

NMR spectroscopy
^{1}H and ^{13}C NMR spectra were recorded on Avance III HD 400 or Bruker Avance III HD 600 spectrometers. Chemical shifts *δ* are given in ppm and *J* (coupling constants) in Hz. For all multiplicities, the following abbreviations were used: s = singlet, d = doublet, dd = doublet of doublet, m = multiplet. Residual solvent signals were used for calibration: ^{1}H NMR: in $CDCl_3$ δ = 7.26 ppm, in CD_2Cl_2 δ = 5.30 ppm; ^{13}C NMR: in CD_2Cl_2 δ = 53.52 ppm.

Mass spectrometry
MALDI-TOF mass spectra were recorded on a MALDI-TOF MS ultrafleXtreme from Bruker Daltonics GmbH in positive-ion mode using *trans*-2-[3-(*tert*-butylphenyl)-2-methyl-2-propenylidene]malo-nonitrile (DCTB) as the matrix.

UV/Vis absorption spectroscopy
UV/Vis absorption spectra were measured with a V-770 spectrophotometer (Jasco) equipped with a JASCO PAC-743R Auto Peltier 6/8-cell changer system for temperature control. Standard quartz glass cuvettes of different path lengths (Hellma) and spectroscopy grade solvents (chloroform and 1,1,2,2-tetrachloroethane purchased from Acros organics) were used.

Fluorescence spectroscopy
Photoluminescence measurements were performed using an Edinburgh Instruments FLS980 spectrometer. Time-resolved measurements employed either an AGILE picosecond pulsed laser source coupled to the excitation monochromator of the spectrometer or pulsed EPL diode lasers for excitation, with fluorescence decays recorded by time-correlated single-photon counting (TCSPC). All spectra were corrected for the wavelength-dependent photomultiplier tube sensitivity and the spectral intensity profile of the excitation source.

Solution measurements were carried out in quartz cuvettes. For solid-state measurements, ensembles of single crystals were immersed in a minimal amount of mineral oil and mounted on quartz substrates.

Absolute fluorescence quantum yields were determined with a Hamamatsu Photonics C9920-02 Absolute Photoluminescence Quantum Yield Measurement System, which is composed of a 150 W CW Xenon lamp as the excitation source, a monochromator (250−700 nm, full width at half-maximum (FWHM) 10 nm), a calibrated integrating sphere, and a multichannel spectrometer capable of simultaneously measuring multiple wavelengths between 300 and 950 nm. Absolute fluorescence quantum yield values were determined for each sample as average value for three different excitation wavelengths (260, 280, and 300 nm).

Fluorescence microscopy
Fluorescence microscopy of micrometer-sized single crystals in mineral oil on quartz substrates were recorded with a Zeiss Axio Imager optical polarization microscope equipped with a Hg arc lamp for excitation with UV light.

Synthesis

Vinyl bromide (S1)[S1]

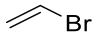

1,2-Dibromoethane (9.60 mL, 21.0 g, 112 mmol) was added to a round bottom flask equipped with a magnetic stirrer and a Y-piece. One end of the Y-piece was equipped with a septum (later with a glass plug), the other one with a micro distillation on top of a condenser. KOH in Ethanol (11.2 g, 1.80 equiv., 200 mmol in 50 mL EtOH) was added via transfer cannulation, where immediate precipitation of KBr and recondensation in the lower part of the condenser happened. The mixture was heated to reflux to distill the product into a cooled flask until no further distillation happened. The product is highly volatile at ambient conditions (bp = 16–20 °C) and was always kept at -10 °C to obtain a colorless liquid, which then was stored in the freezer. The subsequent reaction was carried out within a couple of days to avoid loss and decomposition of this product.

Yield: 10.6 g (99.5 mmol, 89%, Lit.: 88%[S1]) of a colorless liquid.
Boiling Point: 16–20 °C
^{1}H NMR (400 MHz, $CDCl_3$, 295 K): δ = 6.45 (dd, J = 15.3 Hz, J = 7.1 Hz, 1H), 5.99 (dd, J = 7.1 Hz, J = 1.9 Hz, 1H), 5.86 (dd, J = 15.1 Hz, J = 1.9 Hz, 1H) ppm.

1-Ethynyladamantane (1)[S2]

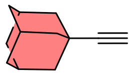

1-Bromoadamantane (14.0 g, 65.0 mmol) was placed into a Schlenk flask under nitrogen atmosphere and cooled to –78 °C. Vinyl bromide (28.0 mL, 41.7 g, 6.00 equiv., 390 mmol, stored in a freezer) was cooled to -78°C and added via transfer cannulation. Afterwards $AlCl_3$ (2.20 g, 0.25 equiv., 16.3 mmol) was added in small portions and the mixture was stirred for 2 h at –78 °C. The mixture was then stirred at –40 °C for 30 min and then at –10 °C for 15 min. The reaction was then cooled again to –40 °C, where first Et_2O (150 mL) and then water (100 mL in small portions, note: exothermic!) were added. The mixture was slowly warmed to room temperature and additional Et_2O (100 mL) and water (50 mL) were added. The layers were separated, and the combined organic layers were washed with brine (50 mL), and dried over Na_2SO_4. After the solvent was removed, the crude intermediate 1-(2,2-dihalogenethyl)adamantane (halogen = Cl, Br) could be obtained as a colorless oil, which was then combined with KOH (30.0 g, 0.54 mol) and dissolved in DMSO (150 mL). The follow up reaction was then stirred with a condenser at 100 °C for 18 h. The reaction was cooled to room temperature and water (250 mL) and Et_2O (100 mL) were added. The phases were separated, and the aqueous phase was extracted with Et_2O (2 × 200 mL). The combined organic layers were washed with water (2 × 100 mL), brine (200 mL) and dried over Na_2SO_4. After the solvent was removed the crude product was purified via distillation/sublimation under reduced pressure (100 °C, ~10^{-2} mbar) into a cooled flask to obtain the product as a white solid.

Yield: 7.40 g (45.5 mmol, 71%, Lit.: 78%[S2]) of a white solid.
Sublimation Point: 100 °C, ~10^{-2} mbar
^{1}H NMR (400 MHz, $CDCl_3$, 295 K): δ = 2.10 (s, 1H), 1.96 (s, 3H), 1.89 (d, J = 3.1 Hz, 6H), 1.70–1.68 (m, 6H) ppm.

1,3,6-Tribromopyrene (6)[S3]

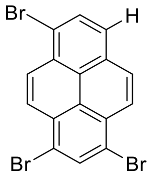

Pyrene (500 mg, 2.47 mmol) and *N*-bromosuccinimide (2.64 g, 6.00 equiv., 14.8 mmol) were dissolved in DMF (100 mL). Afterwards, a few drops of aqueous HBr were added and the reaction was stirred at room temperature overnight. The reaction was then quenched with water (50 mL) and the product was extracted with CH_2Cl_2 (3 × 50 mL). The combined organic phases were washed with water (3 × 50 mL) and dried over Na_2SO_4. After removal of the solvent, the crude product was used for the subsequent reaction without further purification. The ^{1}H NMR data obtained were in agreement with the literature.[S3]

Yield: 803 mg (1.83 mmol, 74%, Lit.: 82%[S3]) of a light yellow solid.

1,6-Bis[(1-adamantyl)ethynyl]pyrene (2)

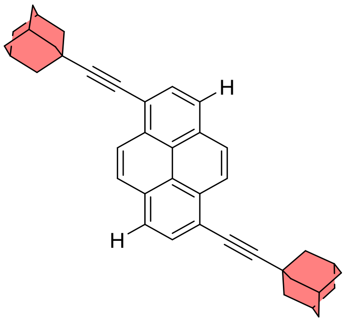

1,6-Dibromopyrene (100.00 mg, 277.74 µmol), 1-ethynyladamantane (178.04 mg, 4.00 equiv., 1.11 mmol), CuI (15.87 mg, 30.0 mol %, 83.32 µmol) and $Pd(PPh_3)_2Cl_2$ (38.99 mg, 20.0 mol %, 55.55 µmol) were charged in a Young Schlenk tube under nitrogen atmosphere. Then degassed triethylamine (5 mL) was added and the mixture was stirred for 3 days at 110 °C. After cooling to room temperature, the mixture was diluted with dichloromethane and remaining solids were filtered off via a short pad of silica-gel. After removal of solvent the crude product was purified by column chromatography (silica-gel, cyclohexane:dichloromethane 9:1) and GPC afterwards. The product was obtained as a white solid.

Yield: 26.5 mg (51.38 µmol, 18%) of a white solid.
Melting point: >300 °C
^{1}H NMR (400 MHz, CD_2Cl_2, 295 K): δ = 8.53 (d, J = 9.1 Hz, 2H), 8.11 (q, J = 8.5 Hz, J = 3.7 Hz, 4H), 8.05 (d, J = 9.1 Hz, 2H), 2.15 (m, 12H), 2.07 (m, 6H), 1.81 (m, 12H) ppm.
^{13}C NMR (151 MHz, CD_2Cl_2, 295 K): δ = 132.18, 130.96, 130.09, 128.20, 126.26, 125.21, 124.55, 119.62, 105.34, 78.45, 43.40, 36.77, 31.09, 28.64 ppm.
HRMS (MALDI-TOF, pos. mode, chloroform/DCTB, 1:3): (m/z) [M]$^+$, calculated for $C_{40}H_{38}$: 518.3000, found: 518.29572
UV/Vis ($CHCl_3$, c = 86 mM): λ_{max} [nm] (ε [L·mol^{-1}·cm^{-1}]) = 392 (83470)

1,3,6-Tris[(1-adamantyl)ethynyl]pyrene (3)

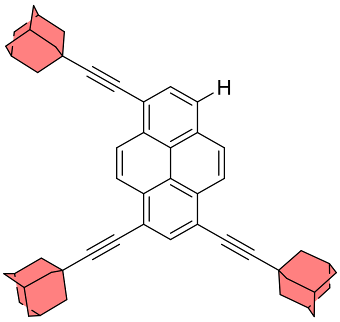

1,3,6-Tribromopyrene (100. mg, 228 µmol), 1-ethynyladamantane (219. mg, 6.00 equiv., 1.37 mmol), CuI (13.0 mg, 30.0 mol %, 68.4 µmol) and $Pd(PPh_3)_2Cl_2$ (32.0 mg, 20.0 mol %, 45.6 µmol) were charged in a Young Schlenk tube under nitrogen atmosphere. Then degassed triethylamine (5 mL) was added and the mixture was stirred for 3 days at 110 °C. After cooling to room temperature, the mixture was diluted with dichloromethane and remaining solids were filtered off via a short pad of silica-gel. After removal of solvent the crude product was purified by column chromatography (silica-gel, cyclohexane:dichloromethane 9:1) and GPC afterwards. The product was obtained as a yellow solid.

Yield: 15.7mg (22.78 µmol, 10%) of a yellow solid.
Melting point: >300 °C
^{1}H NMR (400 MHz, CD_2Cl_2, 295 K): δ = 8.56 (q, J = 18.7 Hz, J = 9.3 Hz, 2H), 8.53 (d, J = 8.9 Hz, 1H), 8.11–8.03 (m, 4H), 2.16–2.13 (m, 18H), 2.07 (m, 9H), 1.80 (m, 18H) ppm.
^{13}C NMR (151 MHz, CD_2Cl_2, 295 K): δ = 131.35, 131.10, 130.26, 128.42, 126.79, 126.42, 126.12, 125.53, 124.56, 124.34, 119.97, 110.34, 105.62, 105.39, 105.33, 78.46, 77.89, 43.40, 43.38, 43.36, 36.76, 36.75, 31.12, 31.08, 31.07, 28.64, 28.63, 28.62 ppm.
HRMS (MALDI-TOF, pos. mode, chloroform/DCTB, 1:3): (m/z) [M]$^+$, calculated for $C_{52}H_{52}$: 676.4100, found: 676.40584
UV/Vis ($CHCl_3$, c = 86 mM): λ_{max} [nm] (ε [L·mol^{-1}·cm^{-1}]) = 413 (84110)

1,3,6,8-Tetrakis[(1-adamantyl)ethynyl]pyrene (4)

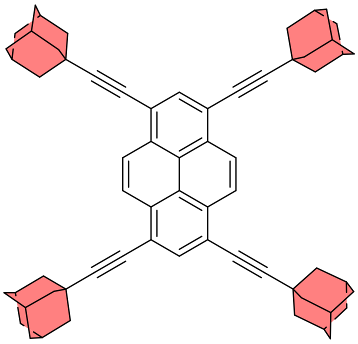

1,3,6,8-Tetrabromopyrene (19.4 mg, 37.4 µmol), 1-ethynyladamantane (60.0 mg, 10.0 equiv., 374 µmol), CuI (2.14 mg, 30.0 mol %, 11.2 µmol) and $Pd(PPh_3)_2Cl_2$ (5.26 mg, 20.0 mol %, 7.49 µmol) were charged in a Young Schlenk tube under nitrogen atmosphere. Then degassed triethylamine (5 mL) was added and the mixture was stirred for 3 days at 110 °C. After cooling to room temperature, the mixture was diluted with dichloromethane and remaining solids were filtered off via a short pad of silica-gel. After removal of solvent the crude product was purified by column chromatography (silica-gel, cyclohexane:dichloromethane 9:1) and GPC afterwards. The product was obtained as an orange solid.

Yield: 13.50 mg (15.72 µmol, 42%) of an orange solid.
Melting point: >300 °C
^{1}H NMR (400 MHz, CD_2Cl_2, 295 K): δ = 8.52 (s, 4H), 8.11 (s, 2H), 2.14 (m, 24H), 2.07 (m, 12H), 1.81 (m, 24H) ppm.
^{13}C NMR (151 MHz, CD_2Cl_2, 295 K): δ = 133.71, 131.51, 126.62, 124.37, 119.64, 105.60, 77.90, 43.36, 36.76, 31.09, 28.62 ppm.
HRMS (MALDI-TOF, pos. mode, chloroform/DCTB, 1:3): (m/z) [M]$^+$, calculated for $C_{64}H_{66}$: 834.5200, found: 834.5159
UV/Vis ($CHCl_3$, c = 86 mM): λ_{max} [nm] (ε [L·mol^{-1}·cm^{-1}]) = 435 (99208)

NMR Spectra

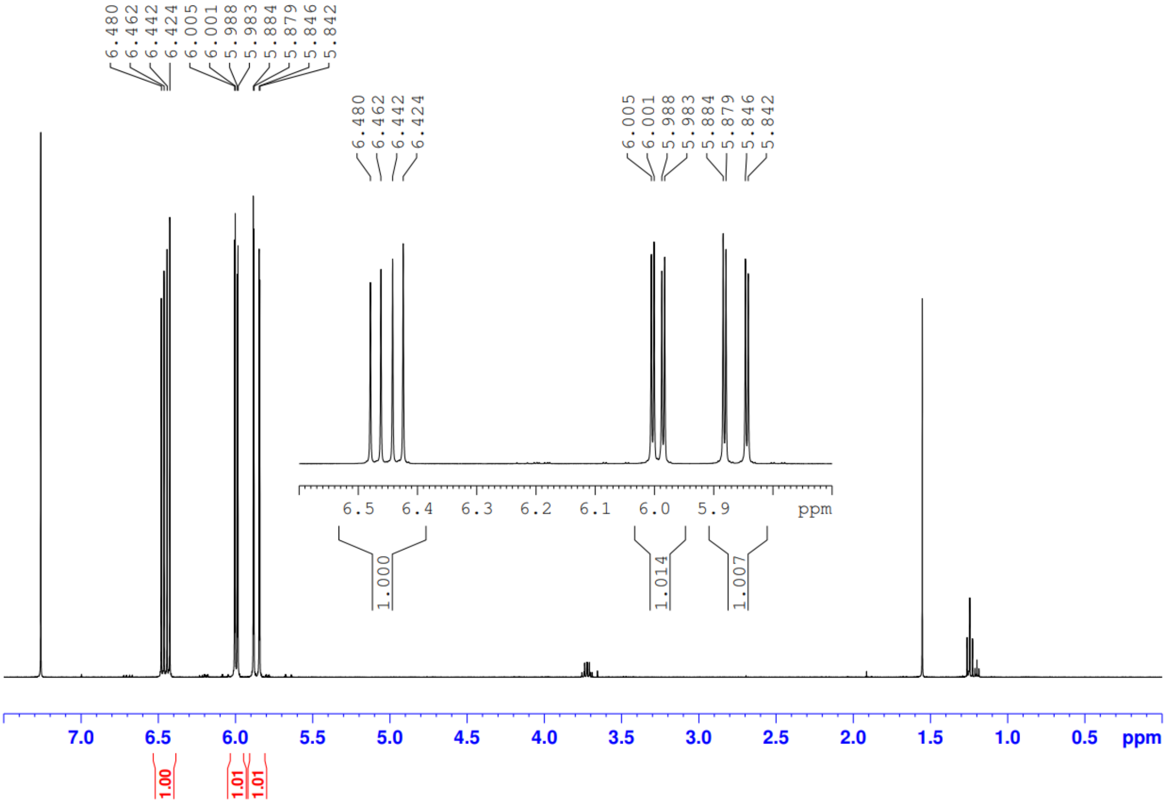

Figure S1. ^{1}H NMR spectrum (400 MHz, 295 K, $CDCl_3$) of **S1**.

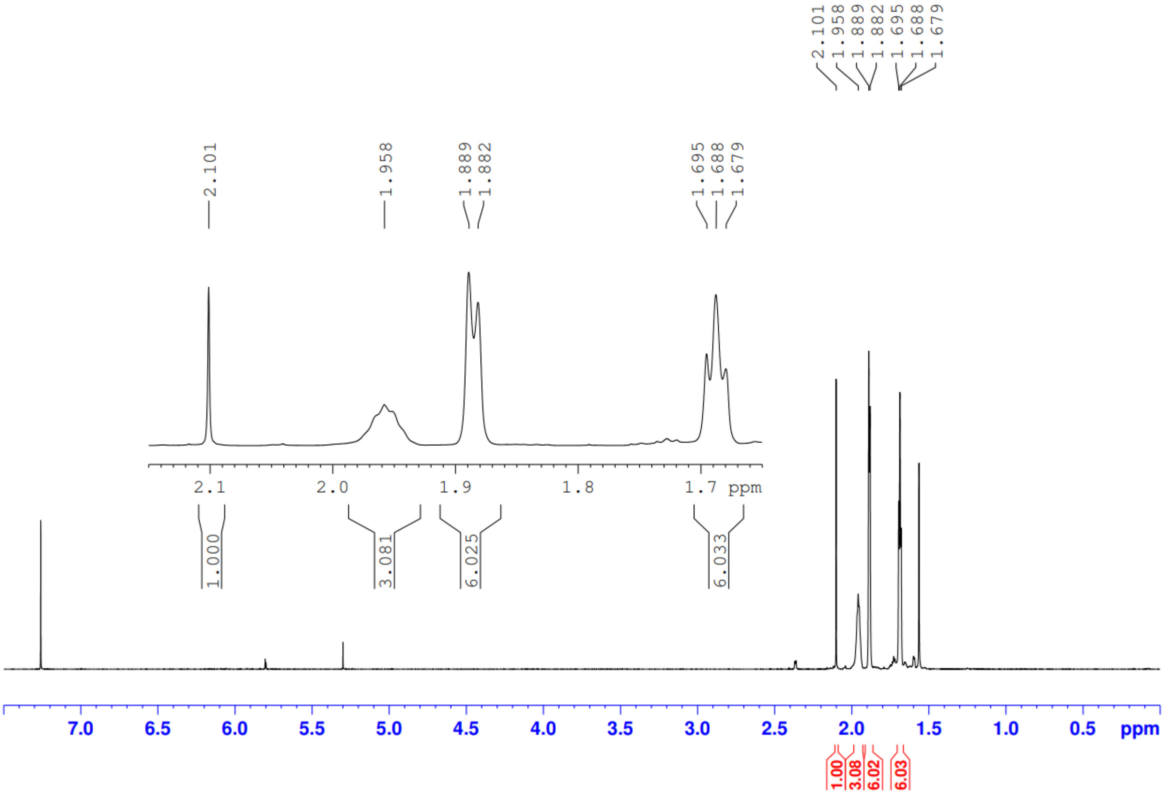

Figure S2. ^{1}H NMR spectrum (400 MHz, 295 K, $CDCl_3$) of **1**.

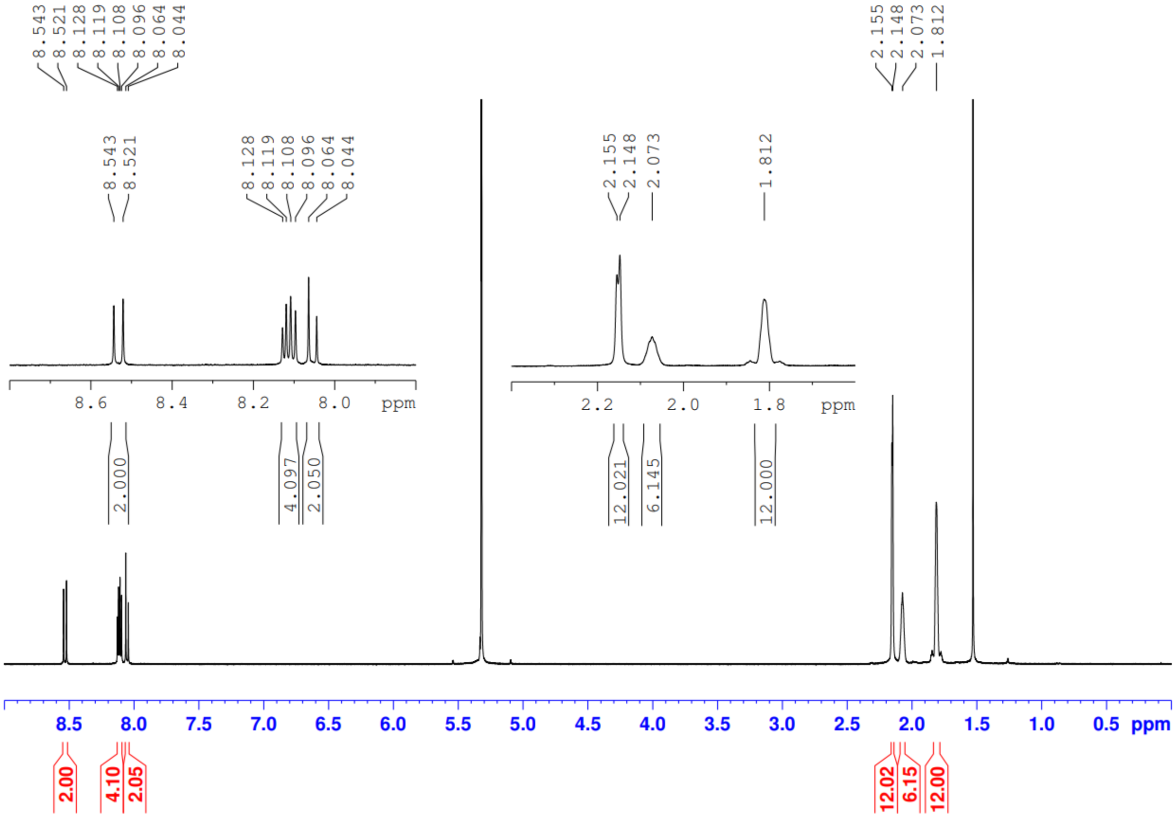

Figure S3. ^{1}H NMR spectrum (400 MHz, 295 K, CD_2Cl_2) of **2**.

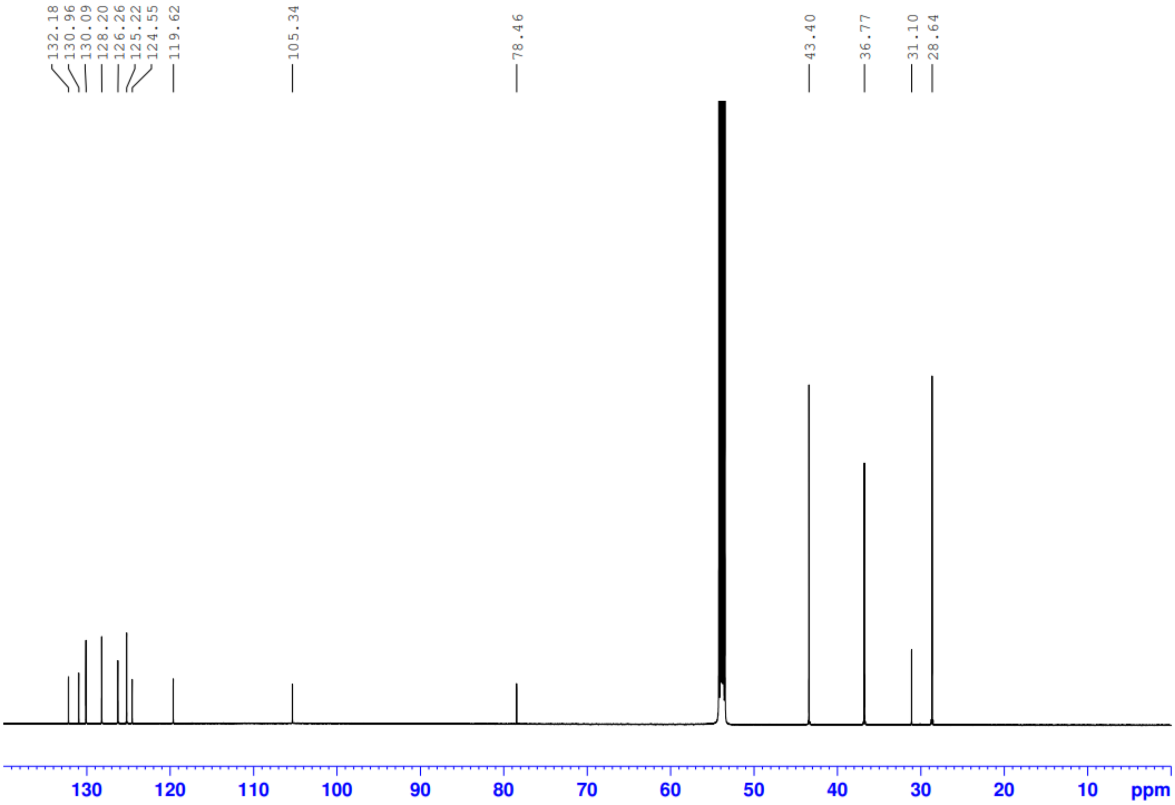

Figure S4. ^{13}C NMR spectrum (151 MHz, 295 K, CD_2Cl_2) of **2**.

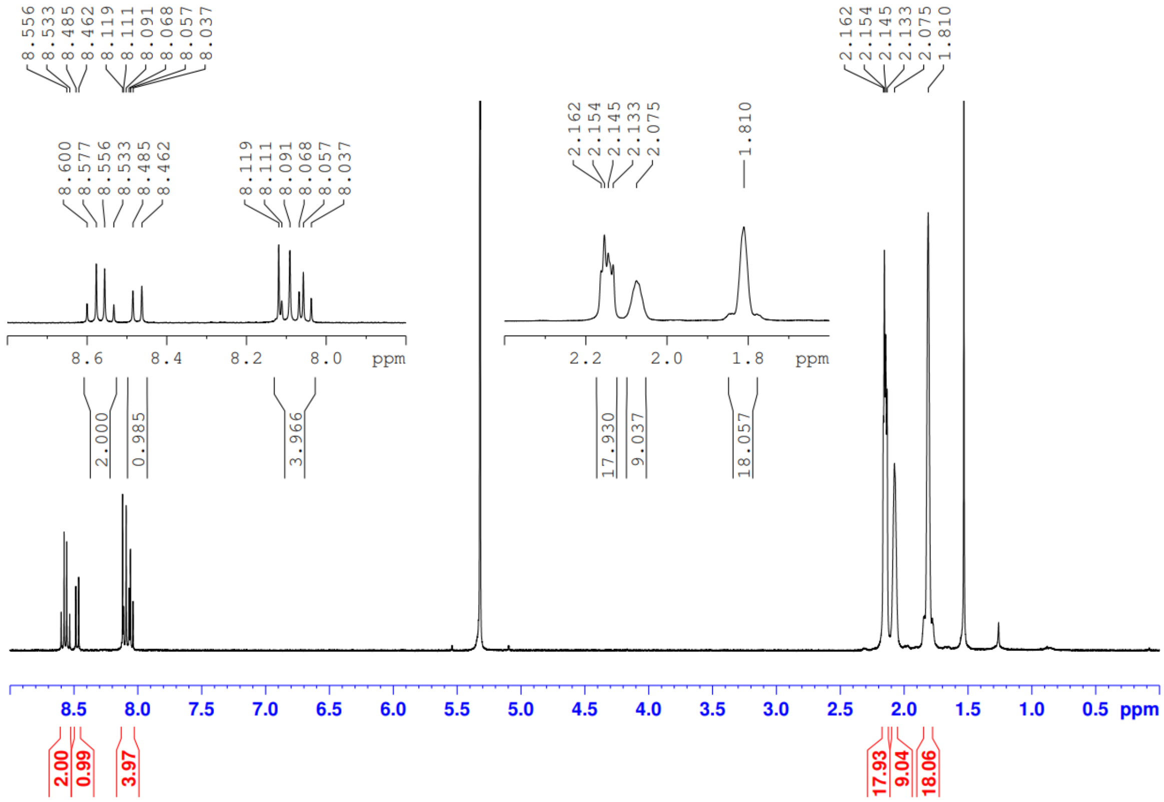

Figure S5. ^{1}H NMR spectrum (400 MHz, 295 K, CD_2Cl_2) of **3**.

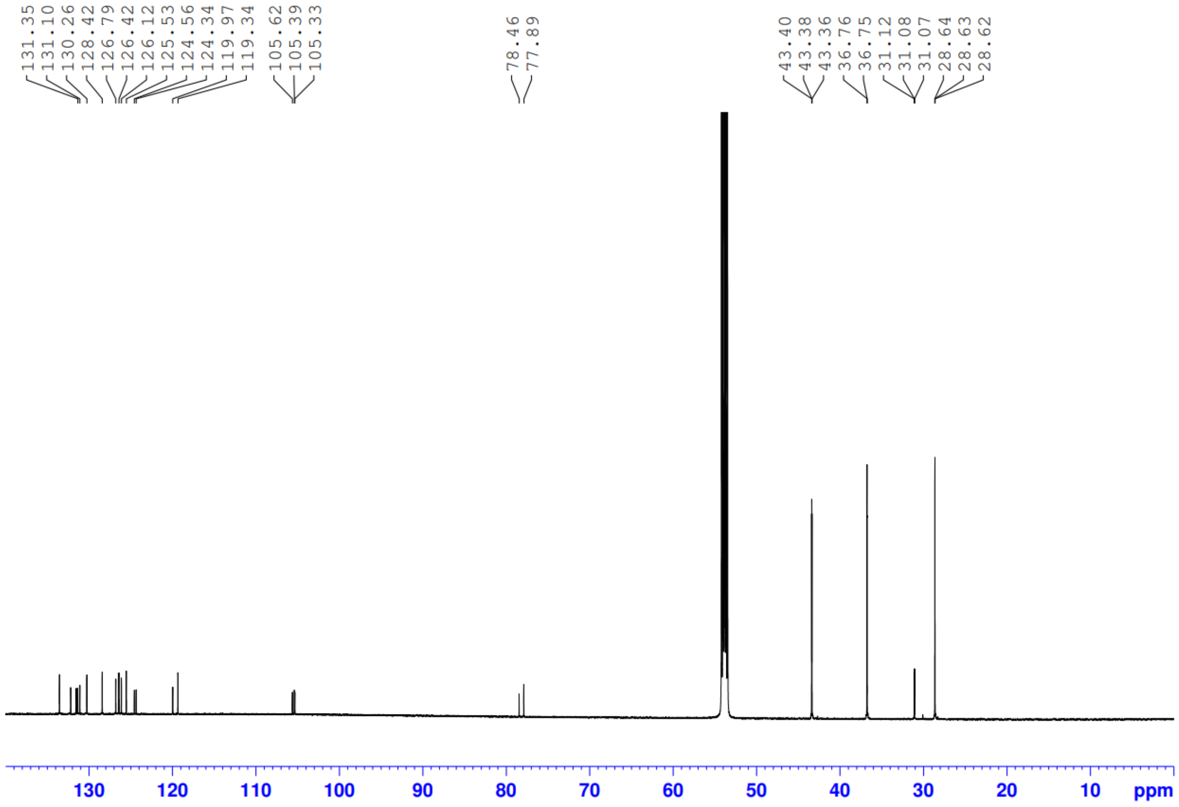

Figure S6. ^{13}C NMR spectrum (151 MHz, 295 K, CD_2Cl_2) of **3**.

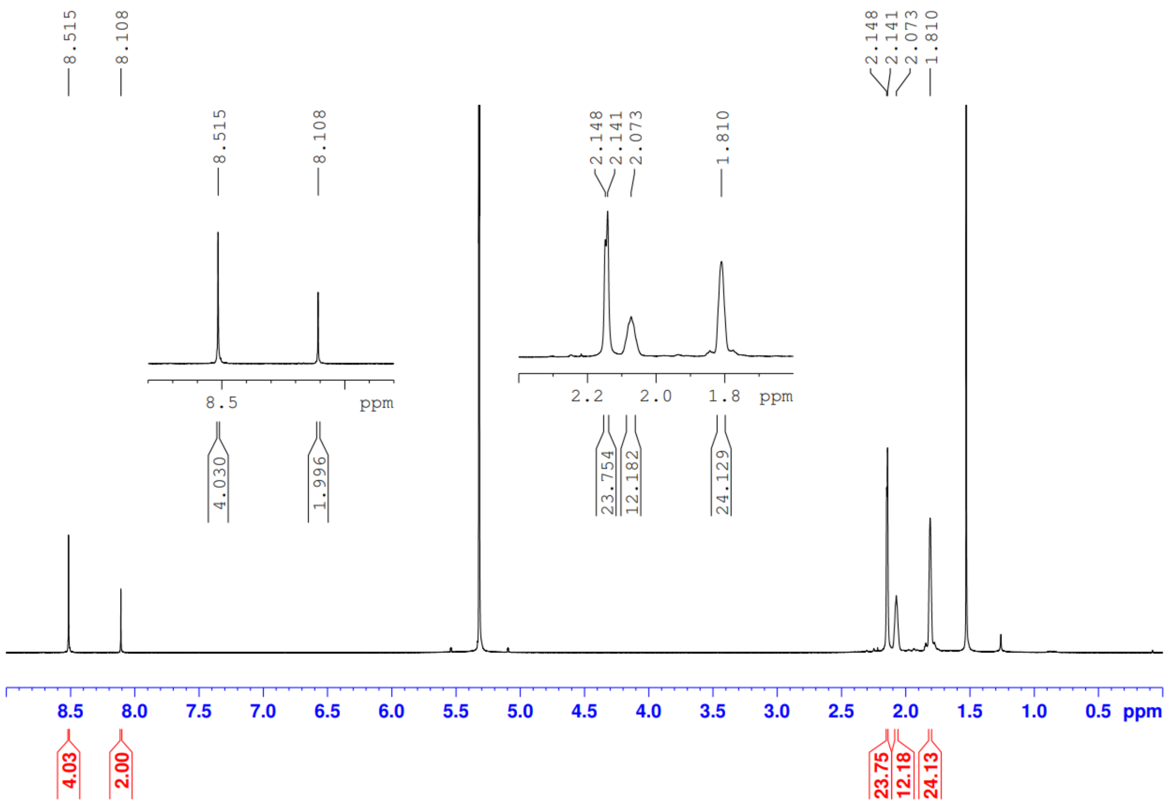

Figure S7. ^{1}H NMR spectrum (400 MHz, 295 K, CD_2Cl_2) of **4**.

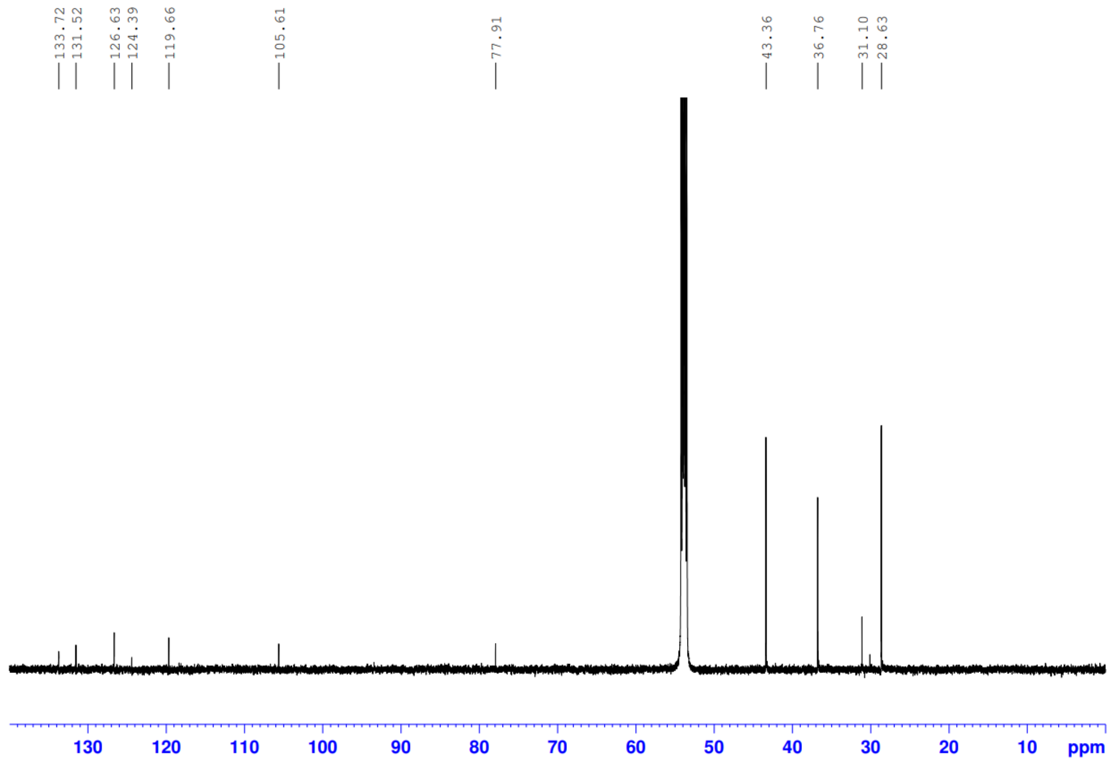

Figure S8. ^{13}C NMR spectrum (151 MHz, 295 K, CD_2Cl_2) of **4**.

Mass Spectra

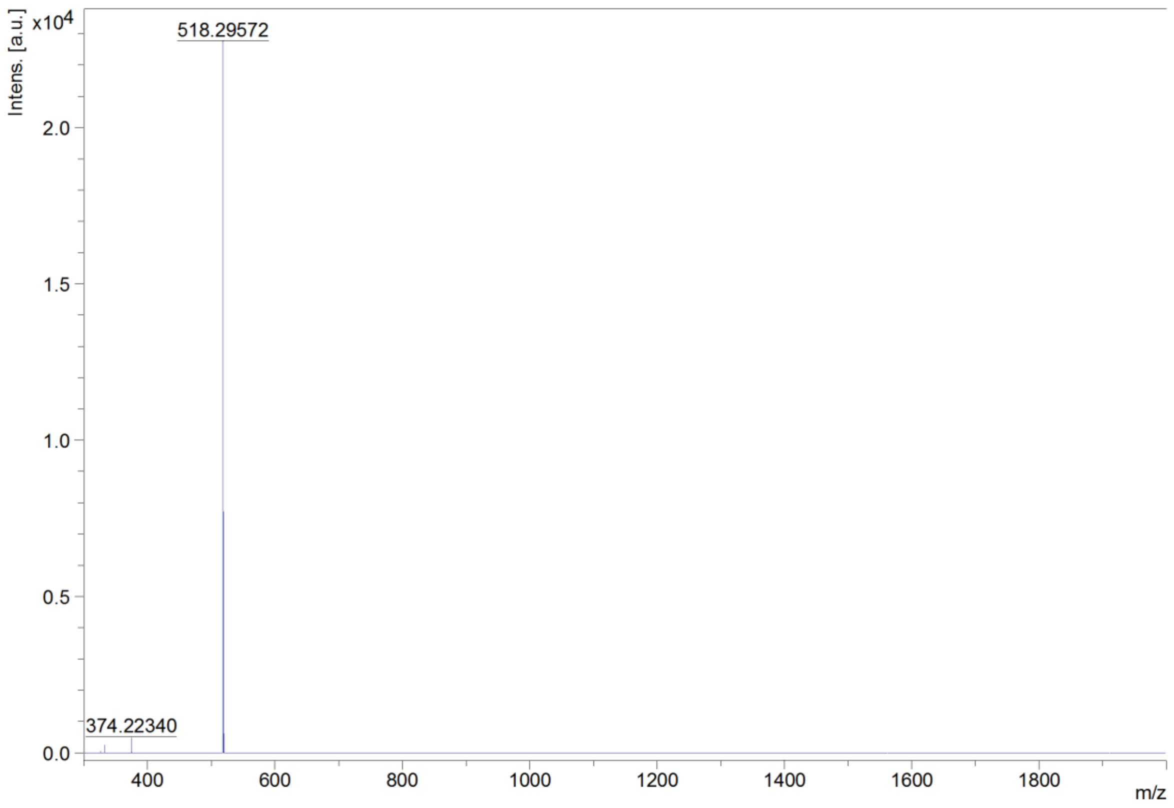

Figure S9. MALDI-TOF mass spectrum of compound **2** in positive-ion mode.

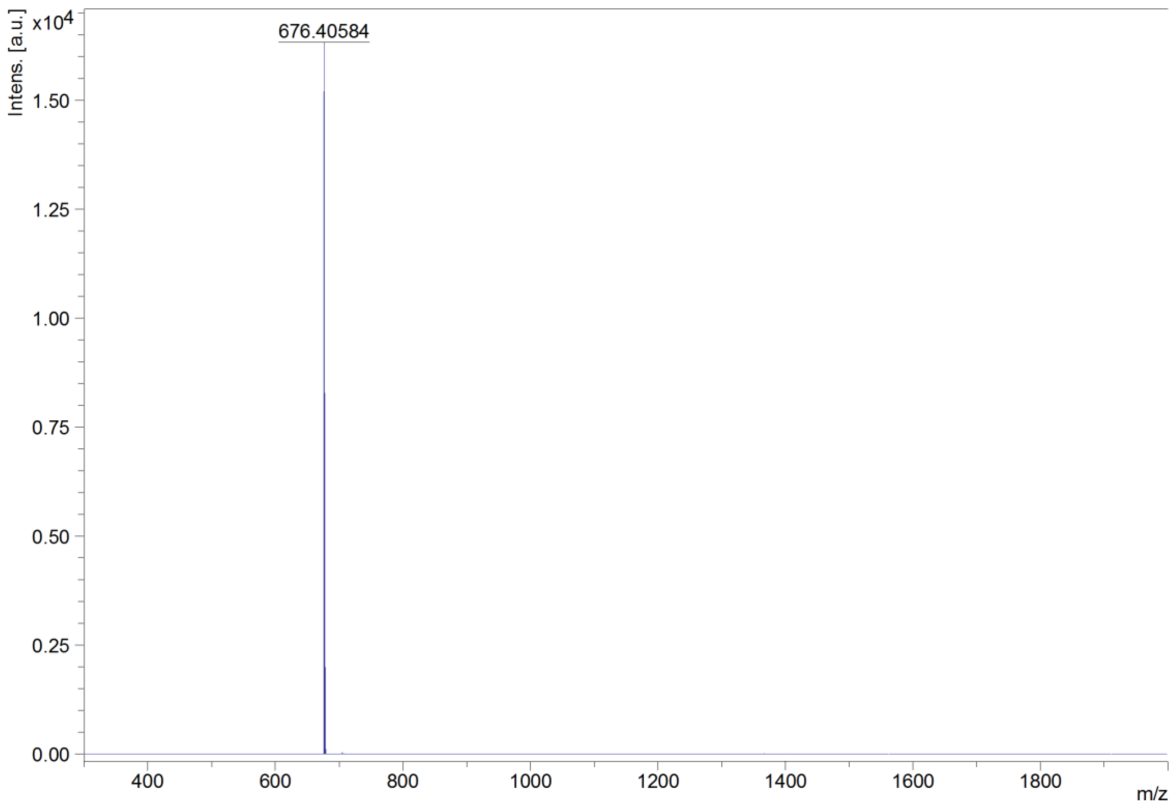

Figure S10. MALDI-TOF mass spectrum of compound **3** in positive-ion mode.

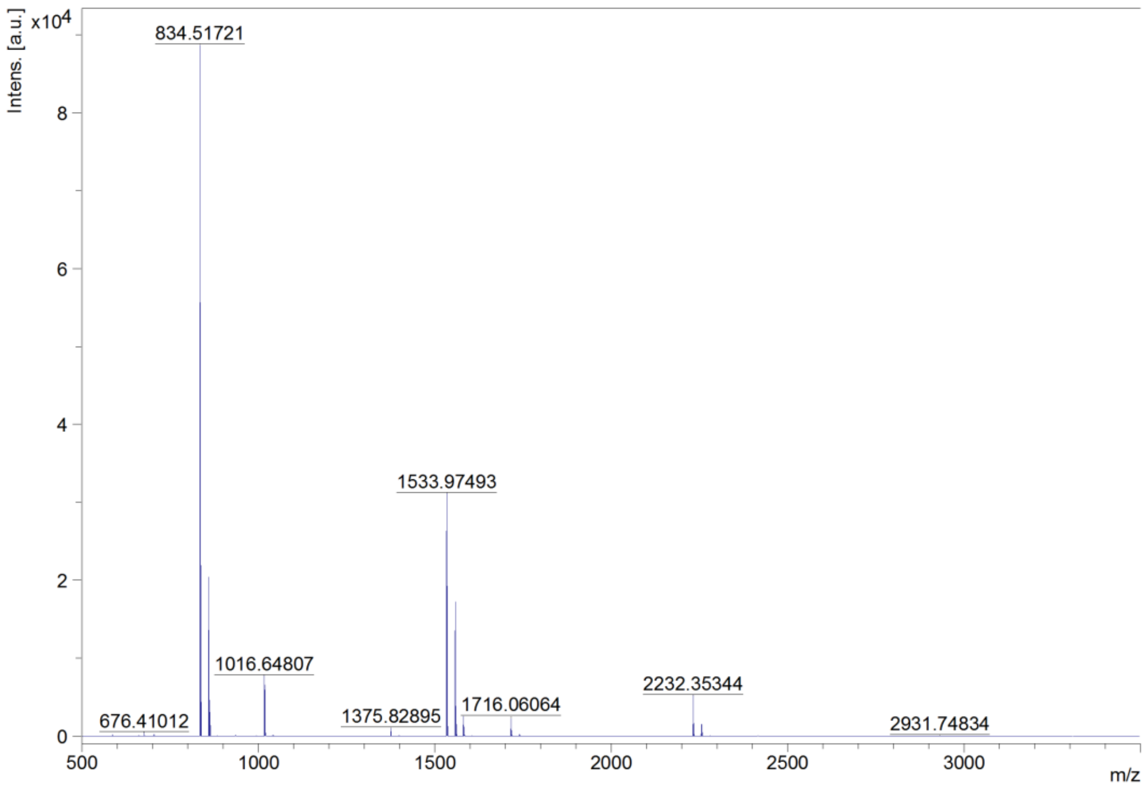

Figure S11. MALDI-TOF mass spectrum of compound **4** in positive-ion mode.

Optical Spectroscopy

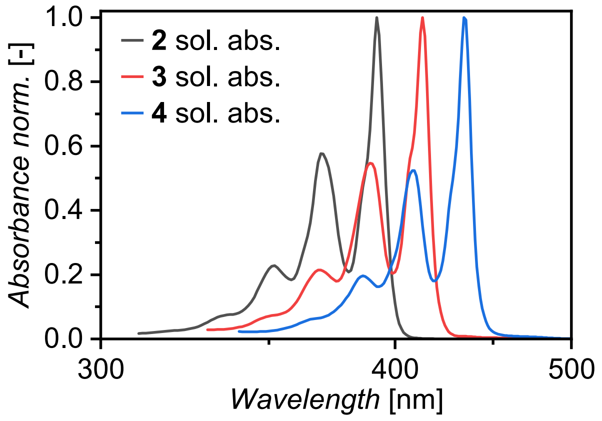

Figure S12. Normalized UV/Vis absorption spectra of **2** (grey), **3** (red), and **4** (blue) in CH_2Cl_2 solution at 295 K.

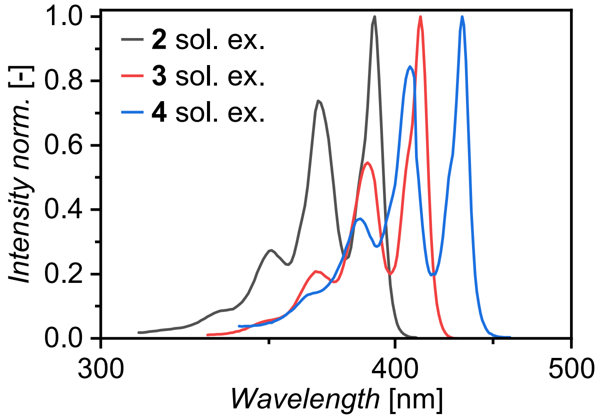

Figure S13. Normalized excitation spectra of **2** (grey), **3** (red), and **4** (blue) in CH_2Cl_2solution at 295 K.

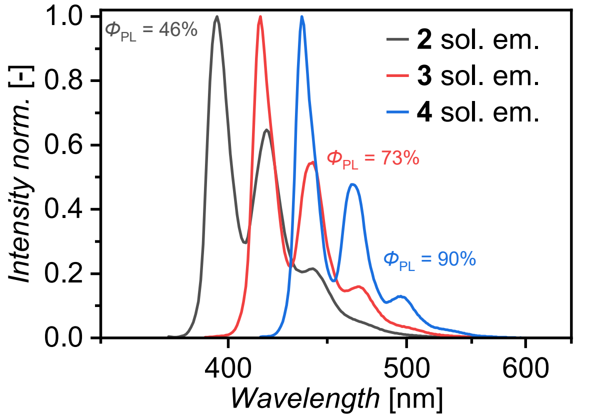

Figure S14. Normalized emission spectra of **2** (grey), **3** (red), and **4** (blue) in CH_2Cl_2 solution at 295 K and the corresponding absolute quantum yields in the respective colors.

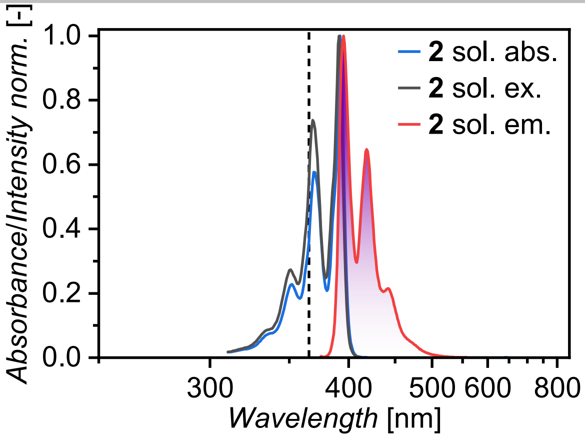

Figure S15. Blue line: normalized absorption spectrum of **2**. Grey line: normalized excitation spectrum of **2**. Red line: normalized emission spectrum of **2**. Area under red line: real emission color. All spectra were measured in CH_2Cl_2 solution at 295 K. The dashed vertical line marks the excitation wavelength for emission spectroscopy at 365 nm.

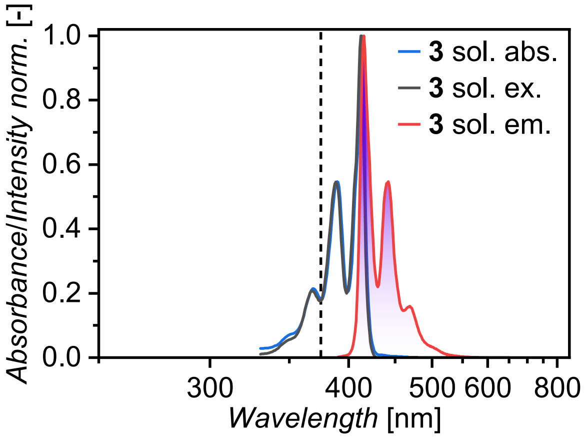

Figure S16. Blue line: normalized absorption spectrum of **3**. Grey line: normalized excitation spectrum of **3**. Red line: normalized emission spectrum of **3**. Area under red line: real emission color. All spectra were measured in CH_2Cl_2 solution at 295 K. The dashed vertical line marks the excitation wavelength for emission spectroscopy at 375 nm.

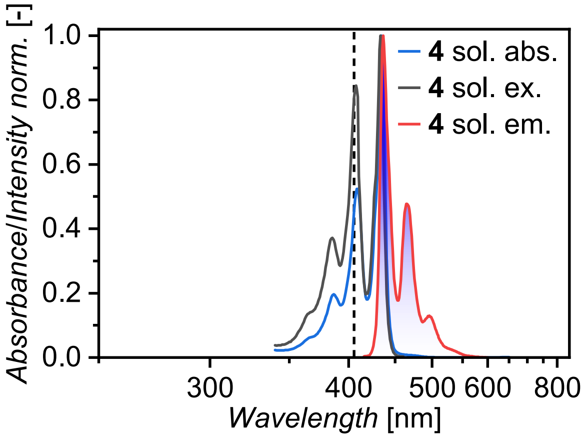

Figure S17. Blue line: normalized absorption spectrum of **4**. Grey line: normalized excitation spectrum of **4**. Red line: normalized emission spectrum of **4**. Area under red line: real emission color. All spectra were measured in CH_2Cl_2 solution at 295 K. The dashed vertical line marks the excitation wavelength for emission spectroscopy at 405 nm.

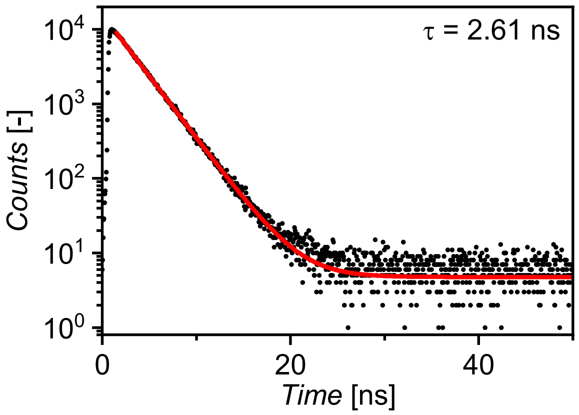

Figure S18. Lifetime measurement of **2** at the 420 nm in CH_2Cl_2 solution at 295 K. The excitation wavelength for this measurement was 378.2 nm. The red line shows the fit of the decay.

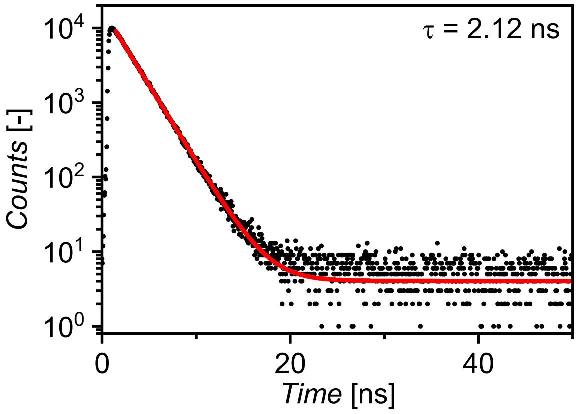

Figure S19. Lifetime measurement of **3** at the 440 nm in CH_2Cl_2 solution at 295 K. The excitation wavelength for this measurement was 378.2 nm. The red line shows the fit of the decay.

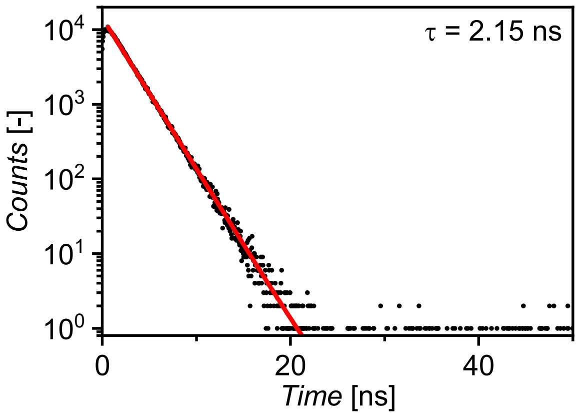

Figure S20. Lifetime measurement of **4** at the 437 nm in CH_2Cl_2 solution at 295 K. The excitation wavelength for this measurement was 406 nm. The red line shows the fit of the decay.

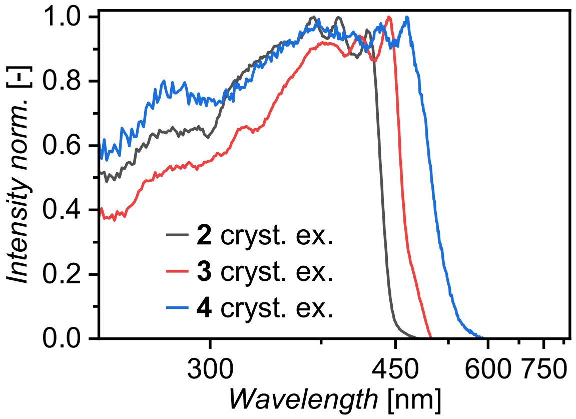

Figure S21. Normalized excitation spectra of crystallites of **2** (grey), **3** (red), and **4** (blue) at 295 K.

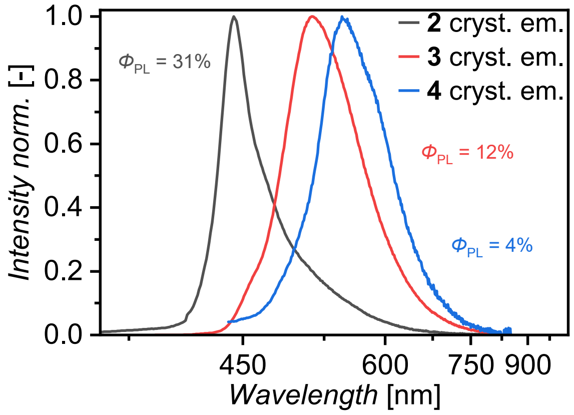

Figure S22. Normalized emission spectra of crystallites of **2** (grey), **3** (red), and **4** (blue) at 295 K and the corresponding absolute quantum yields in the respective colors.

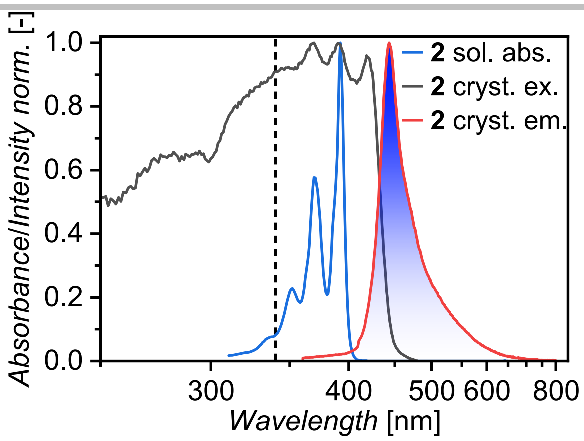

Figure S23. Blue line: normalized absorption spectrum of **2** in CH_2Cl_2 solution. Grey line: normalized excitation spectrum of crystallite ensemble of **2**. Red line: normalized emission spectrum of crystallite ensemble of **2**. Area under red line: real emission color. The dashed vertical line marks the excitation wavelength for emission spectroscopy at 340 nm. All measurements at 295 K.

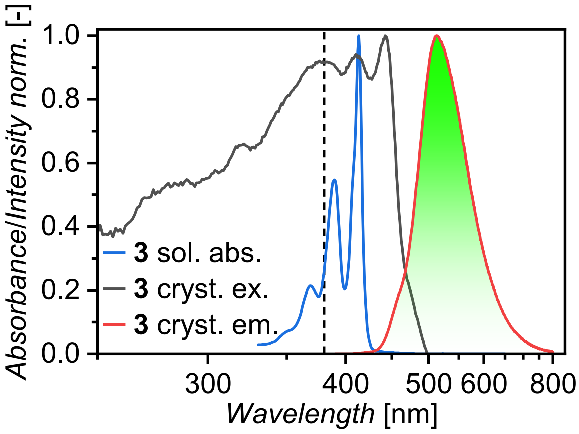

Figure S24. Blue line: normalized absorption spectrum of **3** in CH_2Cl_2 solution. Grey line: normalized excitation spectrum of crystallite ensemble of **3**. Red line: normalized emission spectrum of crystallite ensemble of **3**. Area under red line: real emission color. The dashed vertical line marks the excitation wavelength for emission spectroscopy at 380 nm. All measurements at 295 K.

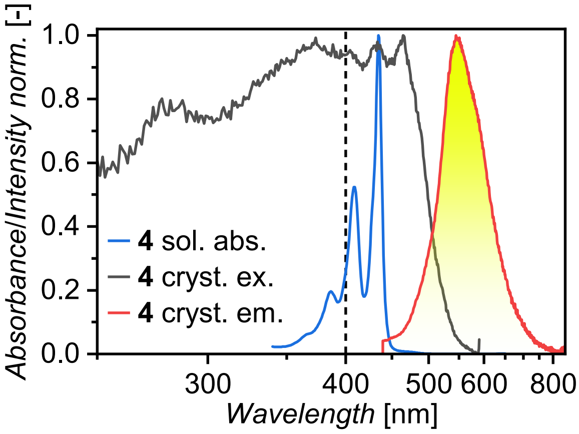

Figure S25. Blue line: normalized absorption spectrum of **4** in CH_2Cl_2 solution. Grey line: normalized excitation spectrum of crystallite ensemble of **4**. Red line: normalized emission spectrum of crystallite ensemble of **4**. Area under red line: real emission color. The dashed vertical line marks the excitation wavelength for emission spectroscopy at 400 nm. All measurements at 295 K.

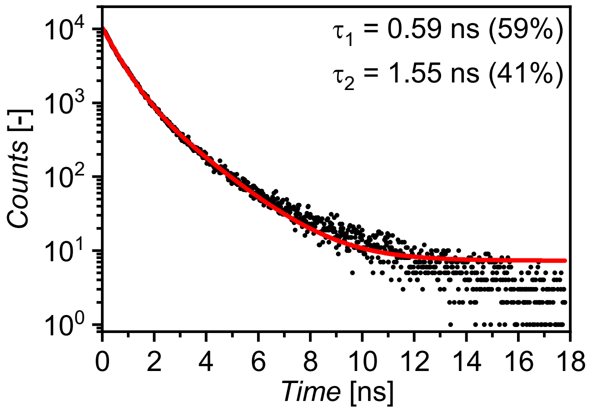

Figure S26. Lifetime measurement of crystallite ensemble of **2** at 445 nm at 295 K. The excitation wavelength for this measurement was 420 nm. The red line shows the fit of the decay.

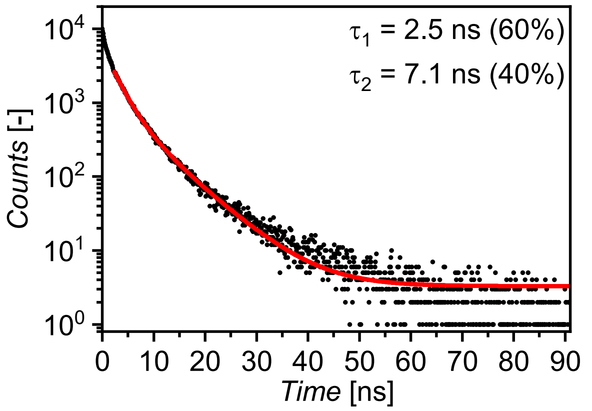

Figure S27. Lifetime measurement of crystallite ensemble of **3** at 515 nm at 295 K. The excitation wavelength for this measurement was 440 nm. The red line shows the fit of the decay.

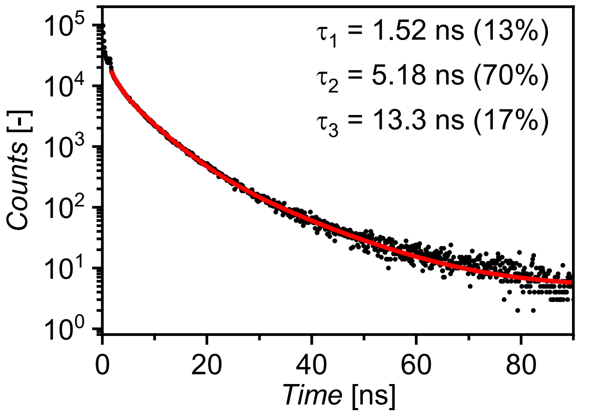

Figure S28. Lifetime measurement of crystallite ensemble of **4** at 546 nm at 295 K. The excitation wavelength for this measurement was 463 nm. The red line shows the fit of the decay.

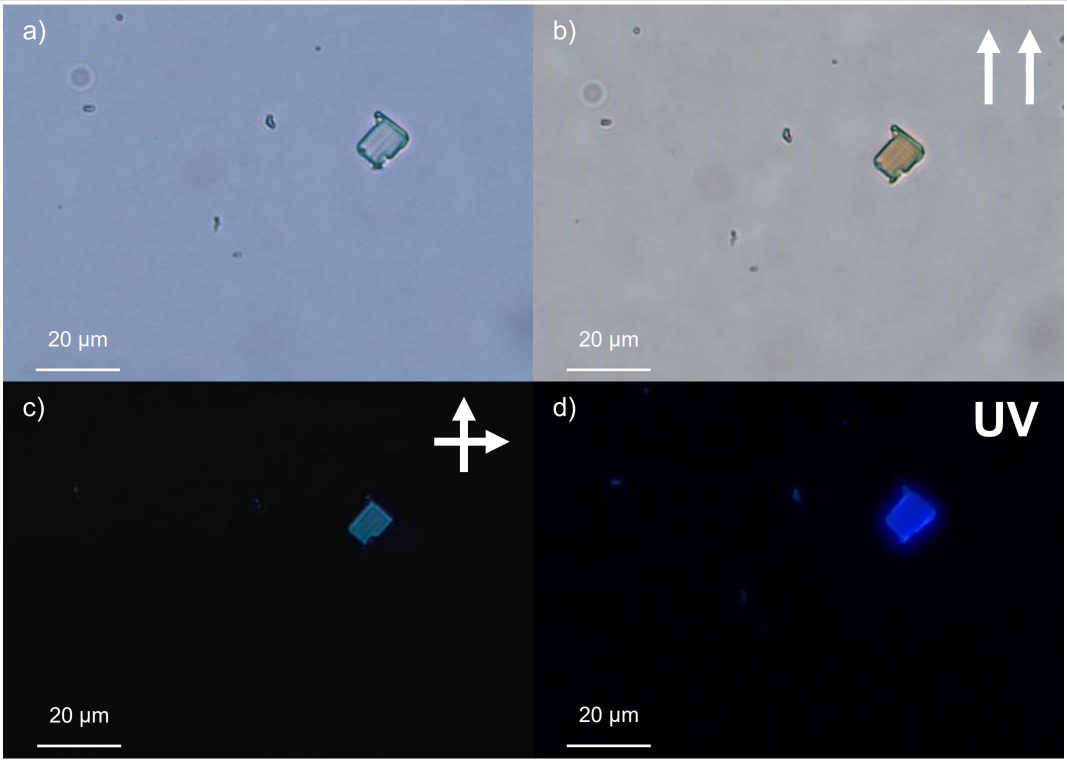

Figure S29. Pictures of a crystal of **2** under a polarization microscope, where **a)** is a picture without polarizer and analyzer, **b)** with polarizer and analyzer both at 0°, and **c)** with the analyzer then at 90°, where a clear double refraction can be seen. **d)** Crystal under UV light with blue emission.

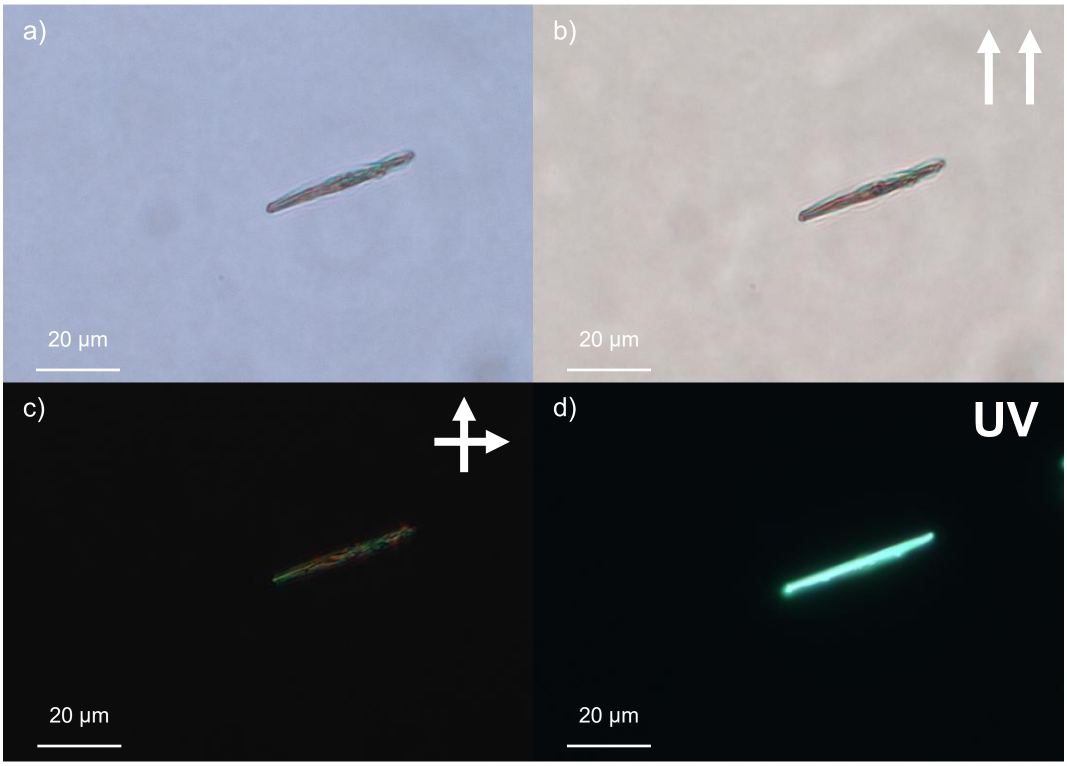

Figure S30. Pictures of a crystal of **3** under a polarization microscope, where **a)** is a picture without polarizer and analyzer, **b)** with polarizer and analyzer both at 0°, and **c)** with the analyzer then at 90°, where a clear double refraction can be seen. **d)** Crystal under UV light with green emission.

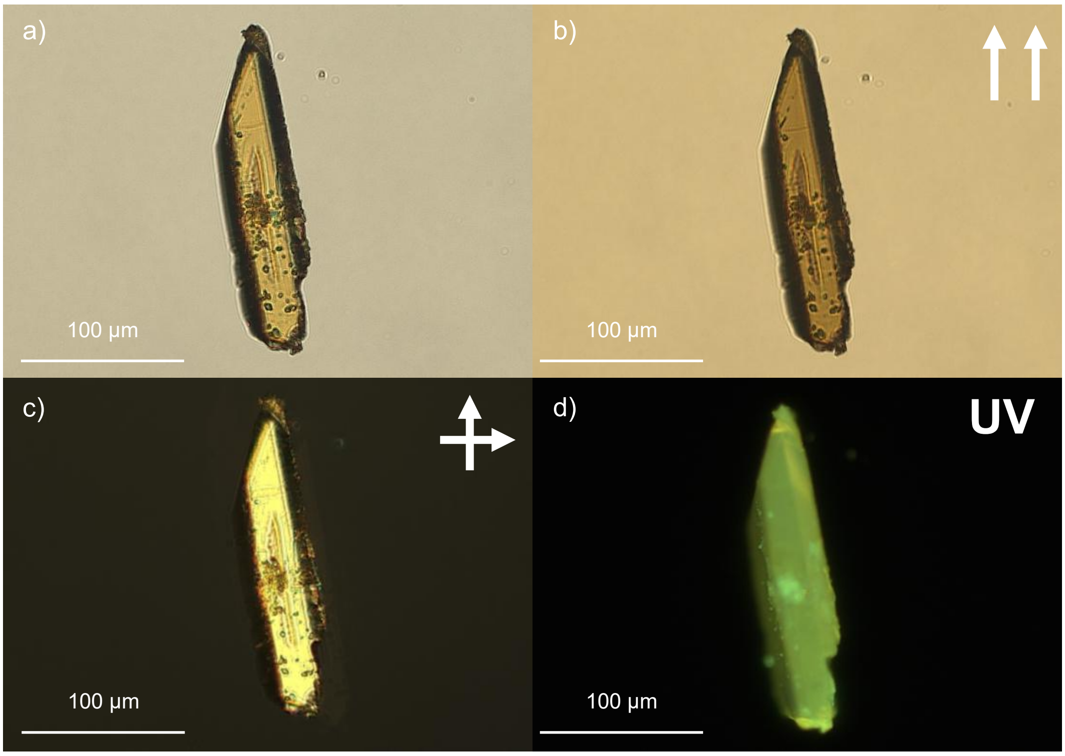

Figure S31. Pictures of a crystal of **4** under a polarization microscope, where **a)** is a picture without polarizer and analyzer, **b)** with polarizer and analyzer both at 0°, and **c)** with the analyzer then at 90°, where a clear double refraction can be seen. **d)** Crystal under UV light with yellow emission.

Table S1. Summary of optical properties of **2**–**4** in their solution state and crystals.[a]

Cmpd.	$\lambda_{abs,s}$	$\lambda_{ex,c}$	$\Delta\lambda_{abs,c\text{-}s}$	$\lambda_{em,s}$	$\lambda_{em,c}$	$\Delta\lambda_{em,c\text{-}s}$
	Solution absorption	Crystal excitation	Solution to crystal shift (absorption and excitation)	Solution emission	Crystal emission	Solution to crystal shift (emission)
**2**	392 nm<br>3.163 eV	419 nm<br>2.962 eV	203 meV	395 nm<br>3.139 eV	443 nm<br>2.800 eV	340 meV
**3**	413 nm<br>3.002 eV	442 nm<br>2.805 eV	196 meV	415 nm<br>2.988 eV	513 nm<br>2.416 eV	571 meV
**4**	434 nm<br>2.857 eV	464 nm<br>2.672 eV	184 meV	436 nm<br>2.844 eV	547 nm<br>2.267 eV	577 meV

[a] Legends: $\lambda_{abs,s}$; lowest energy peak of absorption spectra in dichloromethane at 295 K, $\lambda_{ex,c}$; lowest energy peak of excitation spectra measured for ensemble of crystals at 295 K, $\Delta\lambda_{abs,s\text{-}c}$; solution to crystal shift calculated as $\lambda_{abs,s} - \lambda_{ex,c}$, $\lambda_{em,s}$; highest energy peak of emission spectra in dichloromethane at 295 K. $\lambda_{em,c}$; highest energy peak of emission spectra measured for ensemble of crystals at 295 K. $\Delta\lambda_{em,s\text{-}c}$; solution to crystal shift calculated as $\lambda_{em,s} - \lambda_{em,c}$.

Crystallography

Single crystal X-ray analysis
Single crystal X-ray diffraction data were collected on a Bruker D8 Quest Kappa diffractometer with a Photon II detector and multi-layered mirror monochromated Cu Kα radiation or the P11 beamline at DESY with a single 360 ° φ scan. The diffraction data were indexed, integrated, and scaled using the Bruker APEX4 program suite or XDS program package.[S4] The structures were solved using SHELXT,[S5] expanded with Fourier techniques and refined using the SHELXL software package.[S6] Electron density maps were generated by the ShelXle program[S7] and examined during the analysis. Hydrogen atoms were assigned at idealized positions and were included in the calculation of structure factors. All non-hydrogen atoms in the main residue were refined anisotropically. Disordered side-chains were modelled with restraints using standard SHELXL commands.

For compound **3**, the crystallographic model exhibits level A alerts in the CheckCIF report.[S8] These primarily arise from weak diffraction intensities associated with the large unit cell and pronounced disorder of one of the adamantyl-ethynyl substituents, leading to elevated R values. In addition, the reduced high-angle completeness originates from the single-axis data acquisition strategy employed at P11, combined with outliner rejection during scaling process. Furthermore, the crystal structure of **3** was refined as a twin, identified and treated using the TWINROTMAT routine implemented in PLATON.[S9]

Responses for level A Alerts generated for crystal structure of compound 3

PROBLEM: _diffrn_measured_fraction_theta_full value Low . 0.923 Why?
RESPONSE: The reduced measured fraction of reflections at high theta is attributed to the low diffraction quality of the crystal, which arises from heavy disorder of one of the adamantylethynyl substituents. A significant number of outliers were rejected during scaling due to poor intensity statistics at high resolution.

PROBLEM: High wR2 Value (i.e. > 0.25) .... 0.48 Report
RESPONSE: The elevated wR2 value originates from the low diffraction quality of the crystal, which arises from heavy disorder of one of the adamantylethynyl substituents.
;

Table S2. Crystal data and structure refinement for **2**.

Identification code	CCDC2518021	
Empirical formula	$C_{40}H_{38}$	
Formula weight	518.70	
Temperature	100(2) K	
Wavelength	1.54178 Å	
Crystal system	Monoclinic	
Space group	$P2_1/c$	
Unit cell dimensions	a = 15.896(2) Å	α = 90°
	b = 6.4820(12) Å	β = 91.697(8)°
	c = 13.5800(13) Å	γ = 90°
Volume	1398.6(3) Å^3	
Z	2	
Density (calculated)	1.232 g/cm^3	
Absorption coefficient	0.518 mm^{-1}	
F(000)	556	
Crystal size	0.313 × 0.065 × 0.020 mm^3	
Theta range for data collection	2.78 to 71.71°	
Index ranges	$-19 \le h \le 19$, $-7 \le k \le 8$, $-16 \le l \le 16$	
Reflections collected	12164	
Independent reflections	2706 [R_{int} = 0.0757]	
Completeness to θ = 67.679°	98.8%	
Absorption correction	Semi-empirical from equivalents	
Max. and min. transmission	0.7536 and 0.5526	
Refinement method	Full-matrix least-squares on F^2	
Data / restraints / parameters	2706 / 0 / 181	
Goodness-of-fit on F^2	1.041	
Final R indices [$I > 2\sigma(I)$]	R_1 = 0.0712, wR_2 = 0.1807	
R indices (all data)	R_1 = 0.0879, wR_2 = 0.1973	
Extinction coefficient	n/a	
Largest diff. peak and hole	0.404 and –0.233 e·Å^{-3}	

Table S3. Crystal data and structure refinement for **3**.

Identification code	CCDC2518022	
Empirical formula	$C_{52}H_{52}$	
Formula weight	676.94	
Temperature	100(2) K	
Wavelength	0.72902 Å	
Crystal system	Monoclinic	
Space group	*C2/c*	
Unit cell dimensions	*a* = 22.430(16) Å	*α* = 90°
	b = 6.624(5) Å	*β* = 100.626(13)°
	c = 24.710(18) Å	*γ* = 90°
Volume	3608(5) Å^3	
Z	4	
Density (calculated)	1.246 g/cm^3	
Absorption coefficient	0.073 mm^{-1}	
F(000)	1456	
Crystal size	0.030 × 0.020 × 0.010 mm^3	
Theta range for data collection	1.720 to 28.137°	
Index ranges	$-26 \leq h \leq 25$, $-8 \leq k \leq 8$, $-9 \leq l \leq 31$	
Reflections collected	3186	
Independent reflections	1323 [R_{int} = 0.0820]	
Completeness to θ = 25.939°	92.3%	
Absorption correction	none	
Max. and min. transmission	n/a	
Refinement method	Full-matrix least-squares on F^2	
Data / restraints / parameters	3186 / 187 / 290	
Goodness-of-fit on F^2	1.252	
Final *R* indices [$I > 2\sigma(I)$]	R_1 = 0.1357, wR_2 = 0.4254	
R indices (all data)	R_1 = 0.2193, wR_2 = 0.4821	
Extinction coefficient	n/a	
Largest diff. peak and hole	0.312 and –0.306 e·Å^{-3}	

Table S4. Crystal data and structure refinement for **4**.

Identification code	CCDC2518023	
Empirical formula	$C_{64}H_{66}$	
Formula weight	835.17	
Temperature	100(2) K	
Wavelength	1.54178 Å	
Crystal system	Triclinic	
Space group	$P\bar{1}$	
Unit cell dimensions	a = 6.7036(2) Å	α = 97.8130(10)°
	b = 11.9690(3) Å	β = 97.0190(10)°
	c = 14.9726(4) Å	γ = 106.0730(10)°
Volume	1127.42(5) $Å^3$	
Z	1	
Density (calculated)	1.230 g/cm^3	
Absorption coefficient	0.514 mm^{-1}	
F(000)	450	
Crystal size	0.245 × 0.099 × 0.046 mm^3	
Theta range for data collection	3.022 to 79.376°	
Index ranges	$-8 \leq h \leq 8$, $-15 \leq k \leq 14$, $-18 \leq l \leq 19$	
Reflections collected	22487	
Independent reflections	4725 [R_{int} = 0.0332]	
Completeness to θ = 67.679°	99.7%	
Absorption correction	Semi-empirical from equivalents	
Max. and min. transmission	0.7543 and 0.6164	
Refinement method	Full-matrix least-squares on F^2	
Data / restraints / parameters	4725 / 0 / 289	
Goodness-of-fit on F^2	1.054	
Final R indices [I >2σ(I)]	R_1 = 0.0545, wR_2 = 0.1484	
R indices (all data)	R_1 = 0.0598, wR_2 = 0.1537	
Extinction coefficient	n/a	
Largest diff. peak and hole	0.358 and –0.219 e·$Å^{-3}$	

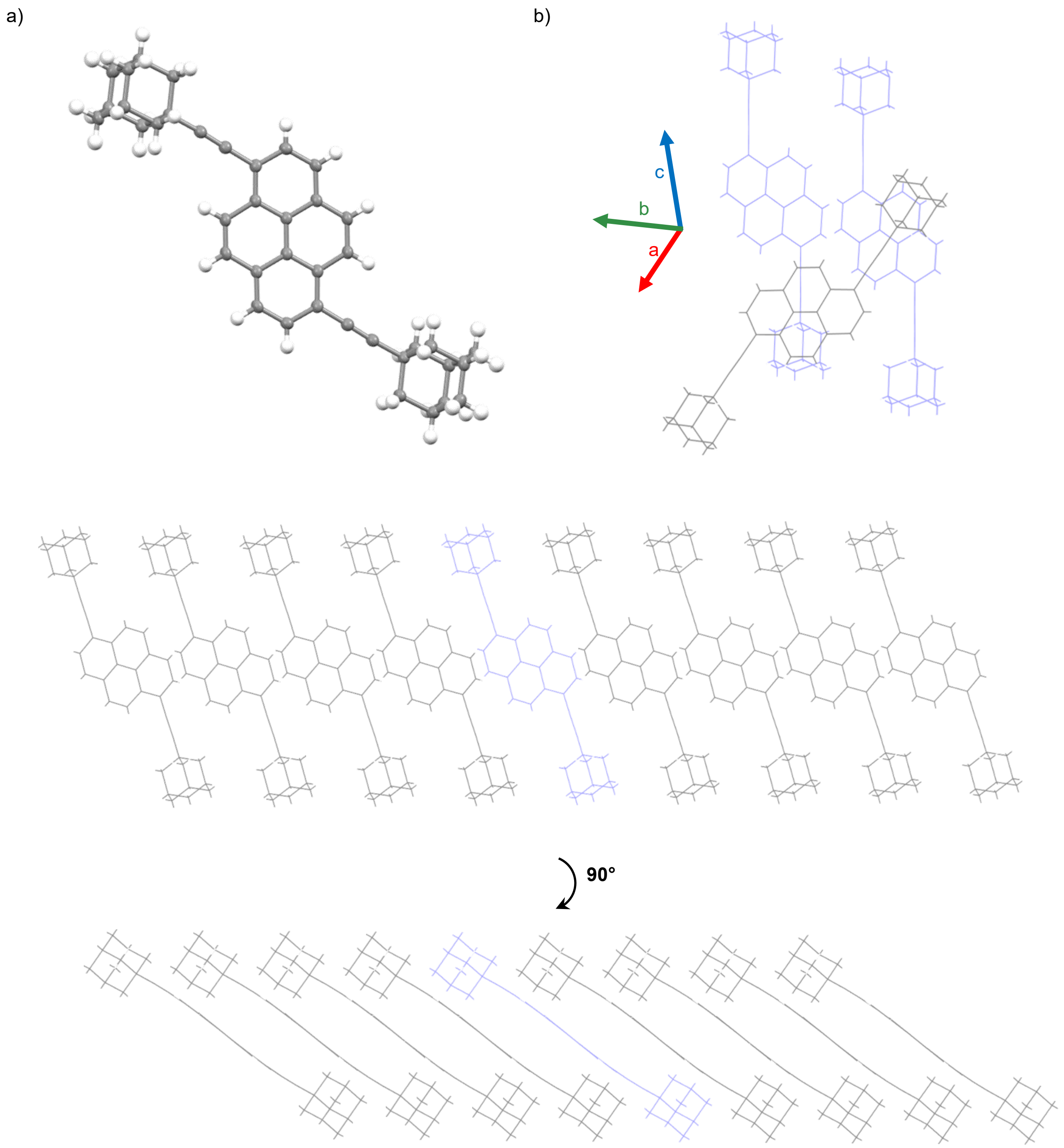

Figure S32. a) Structure drawn by thermal ellipsoids at 50% probability and b) packing drawn in wireframe of compound **2**. One molecule is highlighted in violet for better visualization.

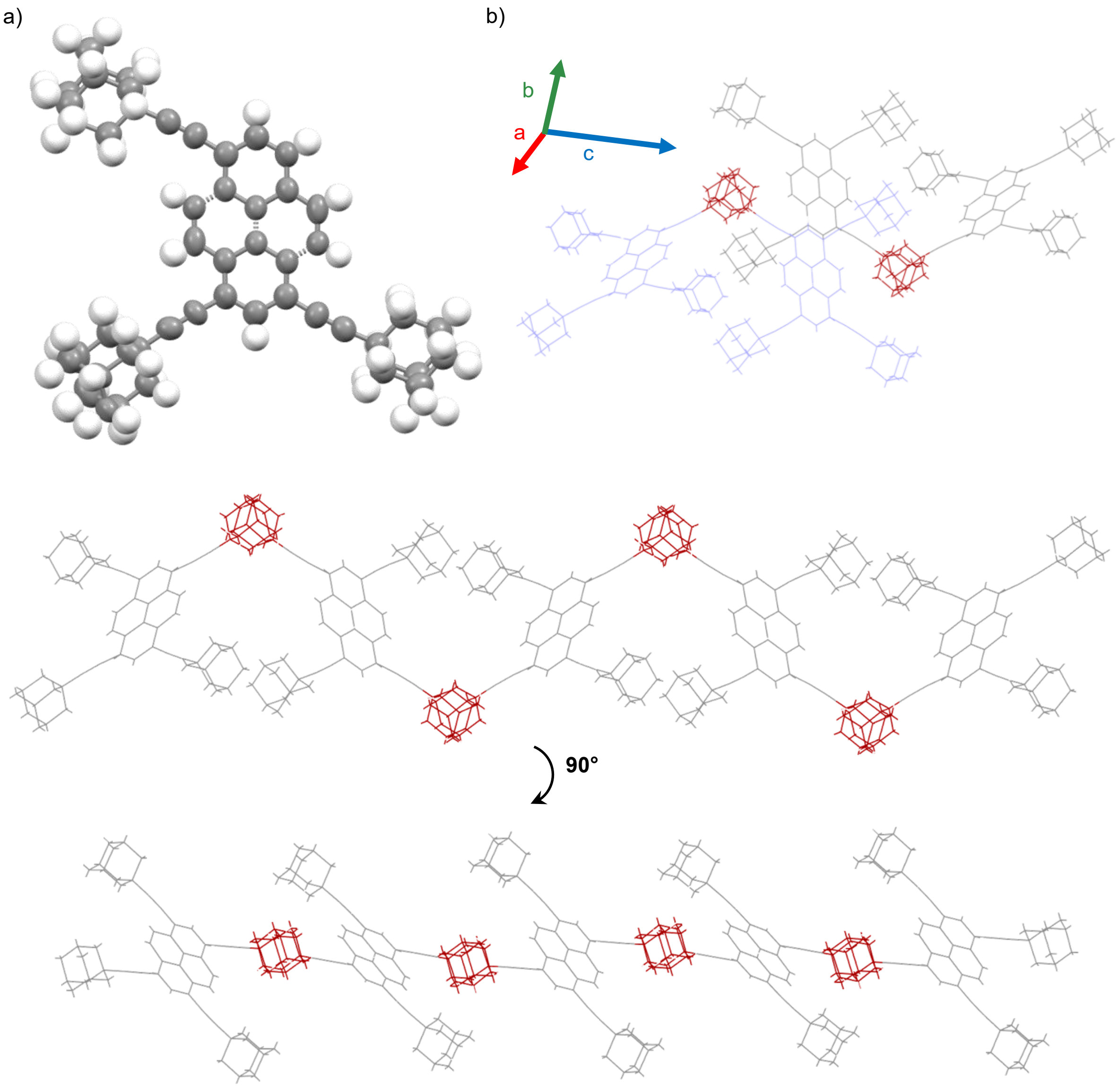

Figure S33. a) Structure drawn by thermal ellipsoids at 50% probability and b) packing drawn in wireframe of compound **3**. The overlappingly shared space of adamantane moieties of the molecule chain is highlighted in red. Two different arrays are visualized with the colors grey and violet.

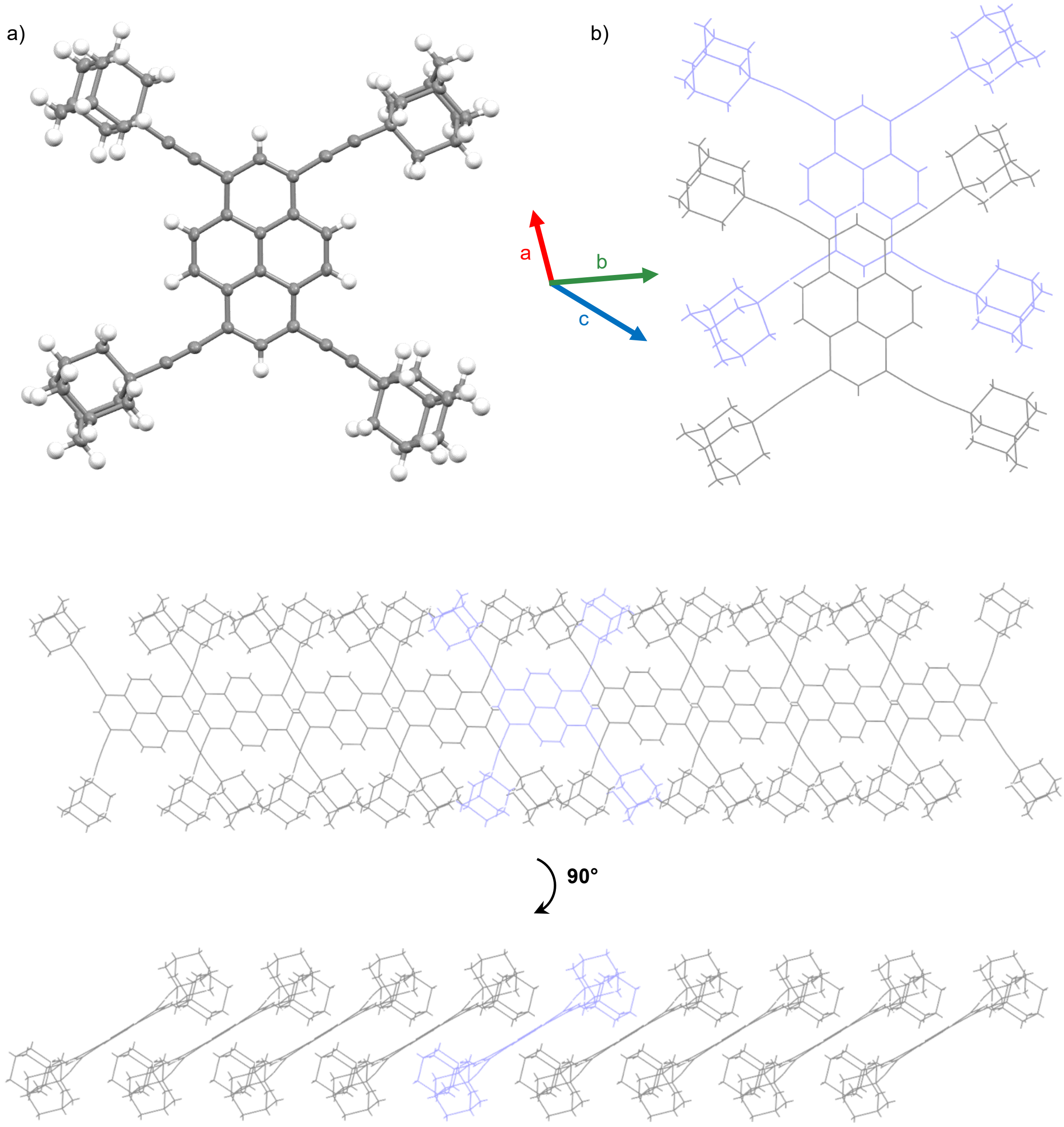

Figure S34. a) Structure drawn by thermal ellipsoids at 50% probability and b) packing drawn in wireframe of compound **4**. One molecule is highlighted in violet for better visualization.

Density Functional Theory (DFT) Calculations

Computational details

Molecular geometries and crystal dimers

Monomer and dimer geometries were taken from the experimental crystal structures. All electronic-structure calculations were performed on these fixed geometries (no further geometry optimization) to ensure direct correspondence to the solid-state packing. For dimer-based analyses (charge-transfer integrals and exciton couplings), molecular pairs were generated directly from the crystallographic coordinates using the relative positions and orientations found in the crystal.

Software

All density functional theory (DFT) and time-dependent DFT (TDDFT) calculations were performed using the Gaussian 16 software package[S10] unless otherwise noted. Constrained DFT (cDFT) calculations for evaluating charge-transfer state energies were carried out using the Q-Chem program package.[S11] Charge transfer integrals between neighboring molecules were calculated using the ADF (Amsterdam Density Functional) software suite employing a fragment-orbital approach.[S 12] Molecular structures, frontier orbital isosurfaces, and schematic orbital representations were visualized using GaussView 6.[S13]

1. Charge-transfer integrals (PW91/TZP)

Charge-transfer (CT) integrals for hole and electron transport, t_{h} and t_{e}, were calculated using density functional theory as implemented in the ADF (Amsterdam Density Functional) software package. All calculations employed the PW91 exchange–correlation functional together with a TZP (triple-ζ plus polarization) basis set. Dimer geometries were constructed directly from the experimental crystal structure, and no geometry optimization was performed in order to preserve the solid-state packing arrangement.

The electronic couplings were evaluated using the fragment-orbital (dimer projection) approach. Monomer frontier orbitals (HOMO for hole transport and LUMO for electron transport) were first calculated for the isolated monomers in the geometries they adopt within the dimer. These orbitals were subsequently used as a localized basis to construct the dimer Hamiltonian and overlap matrices. Effective transfer integrals were obtained from the off-diagonal Hamiltonian and overlap elements, corrected for non-orthogonality by Löwdin orthogonalization according to

$$t = \frac{H_{12} - S_{12}(H_{11} + H_{22})/2}{1 - S_{12}^2}$$

, where $H_{ij}$and S_{ij}denote the Hamiltonian and overlap matrix elements between fragment orbitals i and j.

The relative sign convention of the transfer integrals was determined by visual inspection of the phase relationship of the interacting frontier orbitals. The hole transfer integral was defined such that $t_{\mathrm{h}} > 0$. Following the convention discussed by Spano *et al.* (see, ref. S14), the sign of the hole transfer integral was taken with the opposite sign relative to the corresponding orbital overlap. This reflects the fact that hole transport corresponds to the absence of an electron. Consequently, when HOMO–HOMO and LUMO–LUMO overlaps exhibit the same phase relationship, $t_{\mathrm{h}} > 0$ and $t_{\mathrm{e}} < 0$.

Sign convention and physical interpretation

The relative signs of the hole and electron transfer integrals, t_{h} and t_{e}, have direct implications for the resulting band topology and the nature of exciton coupling. When t_{h} and t_{e} have the same sign, the resulting band structure is J-type. In this case, the valence band exhibits a positive curvature (maximum) and the conduction band a negative curvature (minimum), with both extrema located at the Γ point. This band topology gives rise to a direct lowest-energy vertical optical transition. The associated CT-mediated exciton coupling is likewise J-type (Figure 1b, Figure S38), leading to an optically allowed lowest electronic transition.

In contrast, when t_{h} and t_{e} have opposite signs, the resulting band structure is H-type, characterized by positive curvature (maxima) of both the valence and conduction bands at the Γ point (Figure S39 and Figure S40). In this case, the lowest-energy electronic transition is indirect in momentum space, and the optically allowed transition occurs at higher energy. Nevertheless, the fundamental electronic gap is typically red-shifted relative to the isolated monomer, because the difference $2 \mid t_{\mathrm{e}} - t_{\mathrm{h}} \mid$ leads to an overall narrowing of the band gap. Although the CT-mediated exciton coupling in this case is negative (H-type), the accompanying site-energy correction arising from Frenkel–CT mixing compensates this effect, such that the net CT-mediated contribution still results in a red shift of the optical transition.[S14]

2. Frontier orbitals (HOMO/LUMO) of monomer and dimer (B3LYP/def2svp)

Frontier molecular orbitals of isolated monomers and selected dimers were calculated at the B3LYP/def2svp level of theory. Monomer orbitals were computed on the monomer geometry extracted from the crystal. Dimer orbitals were computed on the full dimer geometry (two monomers in their crystallographic arrangement). Orbital plots shown below were generated from the converged Kohn–Sham wavefunctions using an isovalue of 0.01 a.u. and rendered with Gaussview 6.[S13] Orbital energies are reported relative to the vacuum level as raw Kohn–Sham eigenvalues (not corrected to experimental ionization energies/electron affinities).

3. Coulombic exciton coupling (TrESP; Multiwfn; B3LYP/def2svp)

Coulombic (long-range) Frenkel exciton couplings, J_{Coul}, between symmetry-equivalent molecules were evaluated with the transition charges from electrostatic potentials (TrESP) method.[S15] First, the monomer $S_0 \rightarrow S_1$ transition density (or transition charges) was obtained at the B3LYP/def2svp level on the crystal-derived monomer geometry. Transition charges were then fitted to reproduce the electrostatic potential of the transition density and subsequently used to compute the intermolecular Coulomb interaction energy for each dimer in the crystal geometry:

$$J_{\mathrm{Coul}} = \sum_{A \in 1} \sum_{B \in 2} \frac{q_A^{\mathrm{tr}} q_B^{\mathrm{tr}}}{4\pi\varepsilon_0 R_{AB}}$$

, where q_A^{tr} are the fitted transition charges and R_{AB} are interatomic distances between atoms A and B on different monomers. TrESP fitting and coupling evaluations were carried out with Multiwfn.[S16]

The resulting Coulombic couplings correspond to vacuum values. To approximately account for dielectric screening in the molecular crystal, the calculated couplings were uniformly rescaled by a factor of $1/1.3$, following a literature precedent for organic molecular crystals (ref. S15). This scaling provides an effective correction for medium polarization effects not explicitly included in the vacuum TrESP treatment. All Coulombic exciton coupling values reported in this work refer to these rescaled quantities.

4. CT-mediated exciton coupling (from transfer integrals; E_{S1} by TDDFT; E_{CT} by cDFT)

The CT-mediated exciton coupling (J_{CT}) and site-energy correction (Δ_{CT}) were evaluated within the framework of second-order perturbation theory, treating the intermolecular charge-transfer (CT) states as virtual excited configurations that mix with the local Frenkel excitation.[S14] This treatment is valid under the condition that the electronic coupling matrix elements, given by the electron and hole transfer integrals t_{e} and t_{h}, are small compared to the energy separation between the Frenkel and CT states, i.e. $|t_{\mathrm{e}}|, |t_{\mathrm{h}}| \ll |E_{\mathrm{CT}} - E_{\mathrm{S}_1}|$. Under this condition, direct population of CT states is negligible and their influence on the optically active Frenkel excitation can be accurately described by second-order energy corrections.

The energy of the bright local excitation, E_{S_1}, was determined by TDDFT at the B3LYP/def2svp level on the crystal-derived monomer geometry. The CT-state energy, E_{CT}, was estimated using constrained DFT (cDFT) at the ωB97XD/def2svp level of theory on the corresponding dimer geometry. In this procedure, the energy of the neutral dimer $\mathrm{M}^{(0)}\mathrm{M}^{(0)}$ was first calculated without charge constraints, followed by a calculation of the charge-separated configuration $\mathrm{M}^{+}\mathrm{M}^{-}$, in which one molecule carries a positive charge and the other a negative charge. The resulting energy difference defines the diabatic CT-state energy relevant for Frenkel–CT mixing. Within second-order perturbation theory, the CT-mediated exciton coupling between two equivalent Frenkel excitations is given by

$$J_{\mathrm{CT}} = -2\frac{t_{\mathrm{e}}\, t_{\mathrm{h}}}{E_{\mathrm{CT}} - E_{\mathrm{S}_1}}$$

. The factor of 2 originates from two symmetry-equivalent virtual CT pathways contributing to exciton coupling, namely the $t_{\mathrm{e}} \rightarrow t_{\mathrm{h}}$ and $t_{\mathrm{h}} \rightarrow t_{\mathrm{e}}$ processes (electron transfer followed by hole transfer and vice versa). The overall minus sign follows the sign convention introduced by Spano and ensures consistency between the signs of transfer integrals, band dispersion, and exciton coupling.

In addition to exciton coupling, Frenkel–CT mixing gives rise to a second-order site-energy correction to the local excitation energy. In the polymeric (one-dimensional stack) limit, where each molecule interacts with two equivalent nearest neighbors (left and right), this correction is given by

$$\Delta_{\mathrm{CT}} = -2\frac{t_{\mathrm{e}}^2 + t_{\mathrm{h}}^2}{E_{\mathrm{CT}} - E_{\mathrm{S}_1}}$$

. Here, the factor of 2 reflects contributions from virtual CT recombination processes involving both neighboring molecules. In contrast, for an isolated dimer only a single neighbor contributes, and the corresponding site-energy correction would lack this factor. The total CT-mediated shift of the optical transition energy therefore results from the combined influence of the excitonic splitting term J_{CT} and the site-energy correction Δ_{CT}, and depends sensitively on both the magnitude and relative sign of the electron and hole transfer integrals.

Table S5. Summary of electronic-structure parameters derived from DFT calculations for compounds investigated in this work.

Cmpd.	t_e	t_h	$\Delta E_{band,t}$	$E_{H,mono}$	$E_{L,mono}$	$\Delta E_{L-H,mono}$	$E_{H,di}$	$E_{L,di}$	$\Delta E_{L-H,di}$	$\Delta\Delta E_{L-H,di-mono}$	ΔE_{band}	$\Delta\Delta E_{band,c}$
Unit	meV	meV	meV	eV	eV	eV	eV	eV	eV	meV	meV	meV
**2**	+19.0	−65.0	−168	−5.235	−1.895	3.336	−5.091	−1.854	3.241	−95	−190	−22
**3**	+18.5	+59.0	−155	−5.188	−1.968	3.219	−5.037	−1.918	3.122	−97	−194	−39
**4**	+26.1	+49.3	−151	−5.034	−2.036	3.000	−4.900	−1.993	2.901	−99	−198	−47

[a] t_e, t_h: Electron and hole transfer integrals between neighboring molecules calculated at the PW91/TZP level of theory using crystal-derived dimer geometries. Signs are assigned following the convention described above, such that $t_h > 0$, and J-type orbital overlap corresponds to positive $t_e$values. $\Delta E_{band,t}$: Band-gap narrowing originating solely from transfer-integral–induced band dispersion, defined as $2(| t_e | + | t_h |)$, as obtained from a one-dimensional nearest-neighbor tight-binding model with dispersion $E(k) = E_0 + 2t\cos(ka)$(bandwidth $= 4 | t |$ for each band). $E_{H,mono}$, $E_{L,mono}$: HOMO and LUMO energies of the isolated monomer calculated at the B3LYP/def2svp level of theory. $\Delta E_{L-H,mono}$: Monomer HOMO–LUMO gap defined as $E_{L,mono} - E_{H,mono}$. $E_{H,di}$, $E_{L,di}$: HOMO and LUMO energies of the dimer calculated at the B3LYP/def2svp level of theory using crystal-derived geometries. $\Delta E_{L-H,di}$: Dimer HOMO–LUMO gap defined as $E_{L,di} - E_{H,di}$. $\Delta\Delta E_{L-H,di-mono}$: Change in the HOMO–LUMO gap upon dimer formation, defined as $\Delta E_{L-H,di} - \Delta E_{L-H,mono}$. ΔE_{band}: Total band-gap change upon dimer formation, including both band dispersion and orbital reorganization effects, defined as $2\,\Delta\Delta E_{L-H,di-mono}$ (prefactor 2 from the same one-dimensional tight-binding band-edge relation). $\Delta\Delta E_{band,c}$: Orbital reorganization contribution to the band-gap change, defined as $\Delta E_{band} - \Delta E_{band,t}$.

Table S6. Summary of charge-transfer–related energetic parameters and exciton coupling terms derived from DFT calculations for the investigated compounds.

Cmpd.	t_e	t_h	E_{S1}	E_{CT}	E_{CT}–E_{S1}	Δ_{CT}	J_{CT}	J_{Coul}	$\Delta E_{CT+Coul}$	$\Delta E_{exciton}$
Unit	meV	meV	eV	eV	eV	meV	meV	meV	meV	meV
**2**	+19.0	−65.0	3.10	5.00	1.90	-4.8	+1.3	+13.2	+24.2	-33.8
**3**	+18.5	+59.0	3.08	4.04	0.96	-8.0	-2.3	+3.3	-6.0	-19.2
**4**	+26.1	+49.3	2.84	4.03	1.19	-5.2	-2.2	+7.0	-4.4	-23.6

[a] t_e, t_h: Electron and hole transfer integrals between neighboring molecules calculated at the PW91/TZP level of theory using crystal-derived dimer geometries. E_{S_1}: Energy of the lowest bright singlet excited state of the monomer calculated by TDDFT at the B3LYP/def2svp level of theory. E_{CT}: Charge-transfer state energy estimated as the energy difference between the neutral dimer $M^0M^0$and the charge-separated dimer M^+M^-, calculated by constrained DFT at the ωB97XD/def2svp level of theory. $E_{S_1} - E_{CT}$: Energy separation between the local Frenkel excitation and the CT state used in the perturbative evaluation of CT-mediated effects. Δ_{CT}: Site-energy correction arising from Frenkel–CT mixing in the polymeric one-dimensional limit, calculated as $\Delta_{CT} = -2(t_e^2 + t_h^2)/(E_{CT} - E_{S_1})$. J_{CT}: CT-mediated exciton coupling calculated as $J_{CT} = -2t_e t_h/(E_{CT} - E_{S_1})$, where the prefactor 2 arises from two symmetry-equivalent electron–hole transfer pathways. J_{Coul}: Long-range Coulombic exciton coupling calculated using the TrESP method at the B3LYP/def2svp level of theory and uniformly rescaled by a factor of $1/1.3$ to account for dielectric screening effects in the crystal.[S15] $\Delta E_{CT+Coul}$: Overall CT-mediated and Coulombic excitonic contribution including the site-energy correction Δ_{CT}, with signs preserved (allowed transitions), defined as $\Delta E_{CT+Coul} = \Delta_{CT} + 2J_{CT} + 2J_{Coul}$. $\Delta E_{exciton}$: Upper red-shift limit of CT-mediated and Coulombic excitonic interactions, including the site-energy correction Δ_{CT}, defined as $\Delta E_{exciton} = \Delta_{CT} - 2 | J_{CT} | - 2 | J_{Coul} |$.

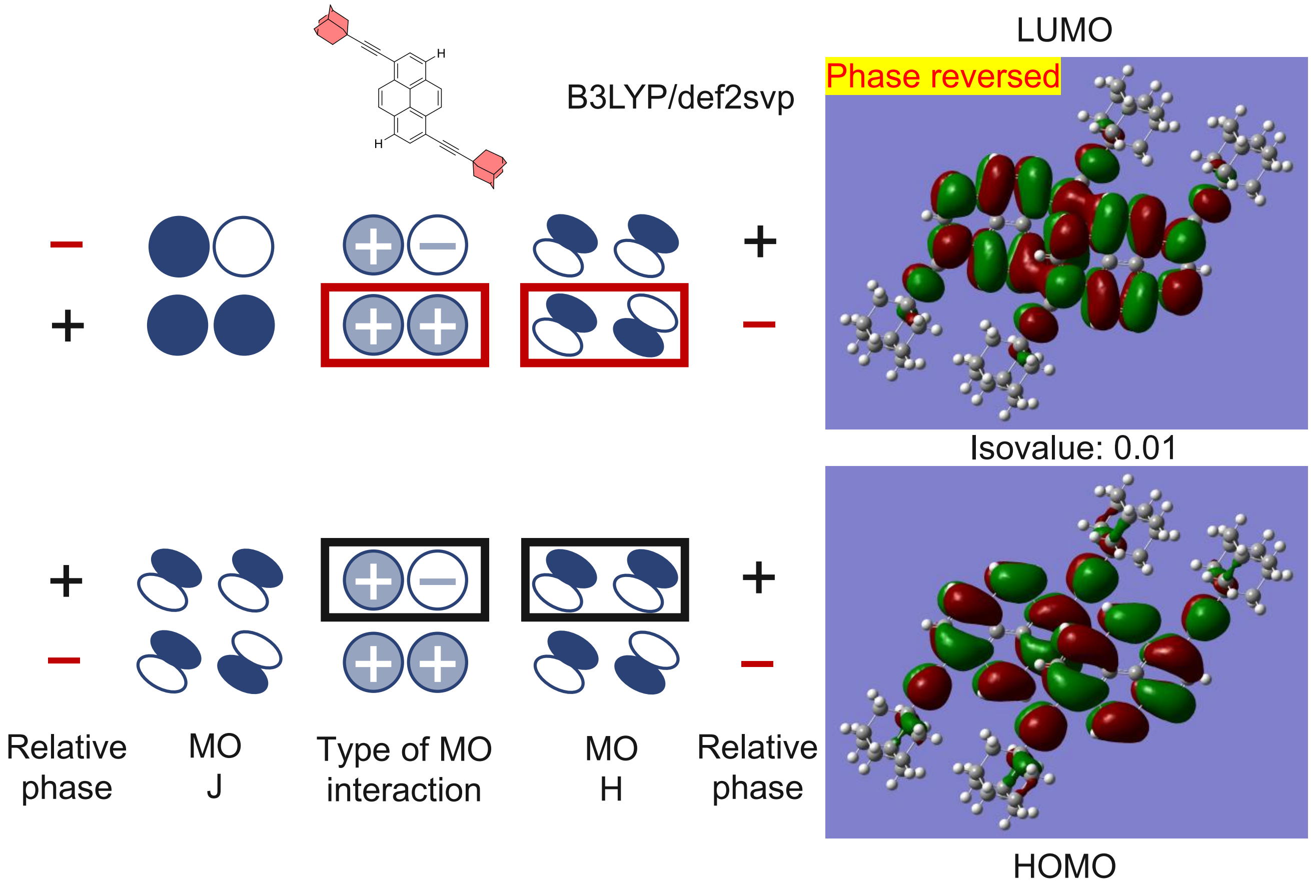

Figure S35. Schematic illustration of the relationship between relative molecular orbital (MO) phase and H-/J-type electronic coupling, together with representative frontier orbital plots for bis(adamantylethynyl)-substituted pyrene **2**. Left: Conceptual depiction of the relative phase of neighboring frontier orbitals and the resulting type of MO interaction. The overall relative phase of the two monomers in a stacked dimer depends on the local orbital interactions and is out-of-phase for HOMO–HOMO interactions (destabilization, higher energy level) and in-phase for LUMO–LUMO interactions (stabilization, lower energy level). For **2**, the overall phase relationship of the two monomers is reversed, as indicated by the black and red boxes, corresponding to H-type charge-transfer coupling ($t_{\mathrm{h}} > 0$, $t_{\mathrm{e}} < 0$). Right: Calculated LUMO (top) and HOMO (bottom) isosurfaces for a representative dimer at the B3LYP/def2svp level of theory (isovalue = 0.01). For the LUMO, the orbital phase is reversed between neighboring molecules relative to the HOMO, leading to opposite signs of the electron and hole transfer integrals (t_{e} and t_{h}).

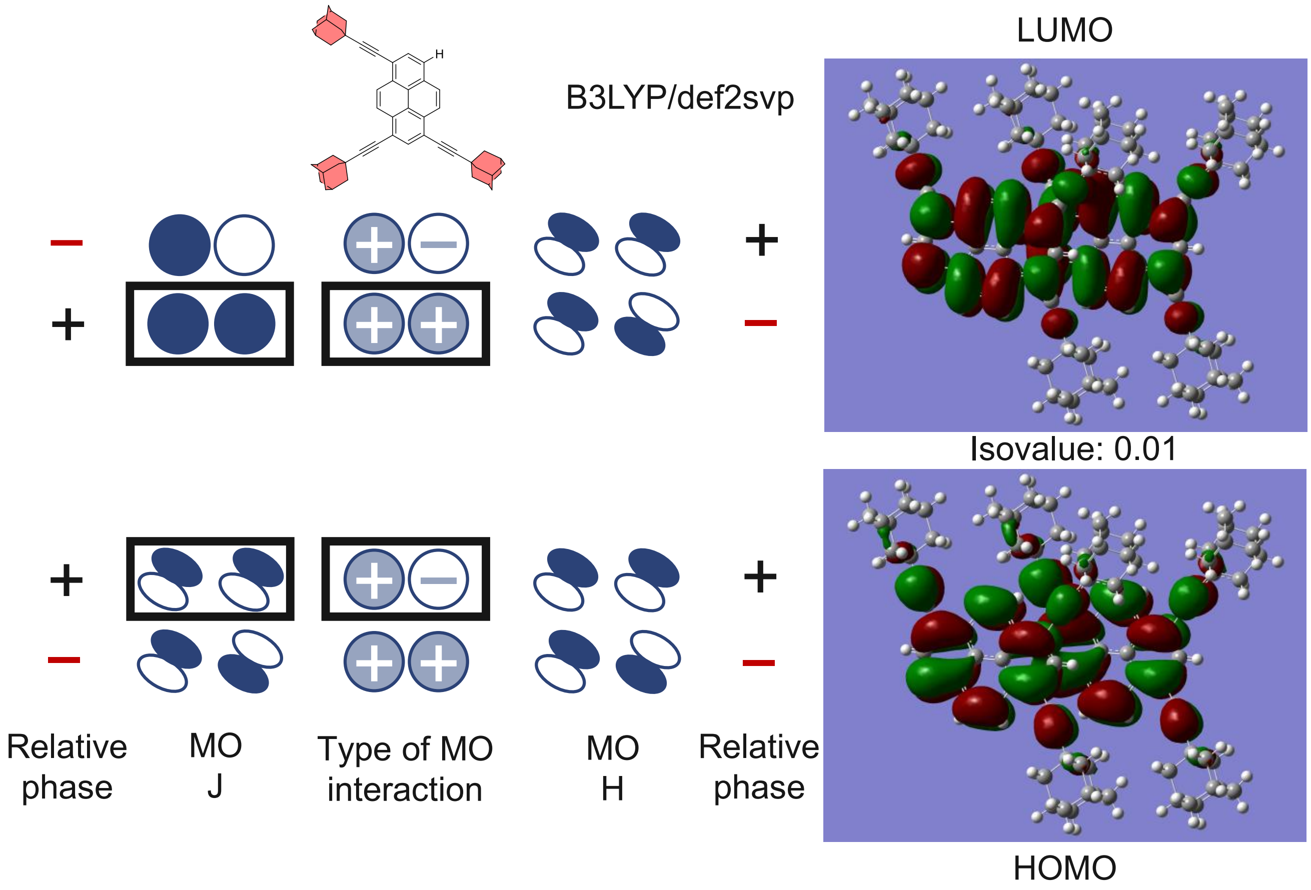

Figure S36. Schematic illustration of the relationship between relative molecular orbital (MO) phase and H-/J-type electronic coupling, together with representative frontier orbital plots for tris(adamantylethynyl)-substituted pyrene **3**. Left: Conceptual depiction of the relative phase of neighboring frontier orbitals and the resulting type of MO interaction. The overall relative phase of the two monomers in a stacked dimer depends on the local orbital interactions and is out-of-phase for HOMO–HOMO interactions (destabilization, higher energy level) and in-phase for LUMO–LUMO interactions (stabilization, lower energy level). For **3**, the overall phase relationship of the two monomers is preserved, as indicated by the black boxes, corresponding to J-type charge-transfer coupling ($t_\mathrm{h} > 0$, $t_\mathrm{e} > 0$). Right: Calculated LUMO (top) and HOMO (bottom) isosurfaces for a representative dimer at the B3LYP/def2svp level of theory (isovalue = 0.01). The consistent phase relationship between neighboring molecules for both frontier orbitals results in transfer integrals of the same sign for electrons and holes (t_e and t_h).

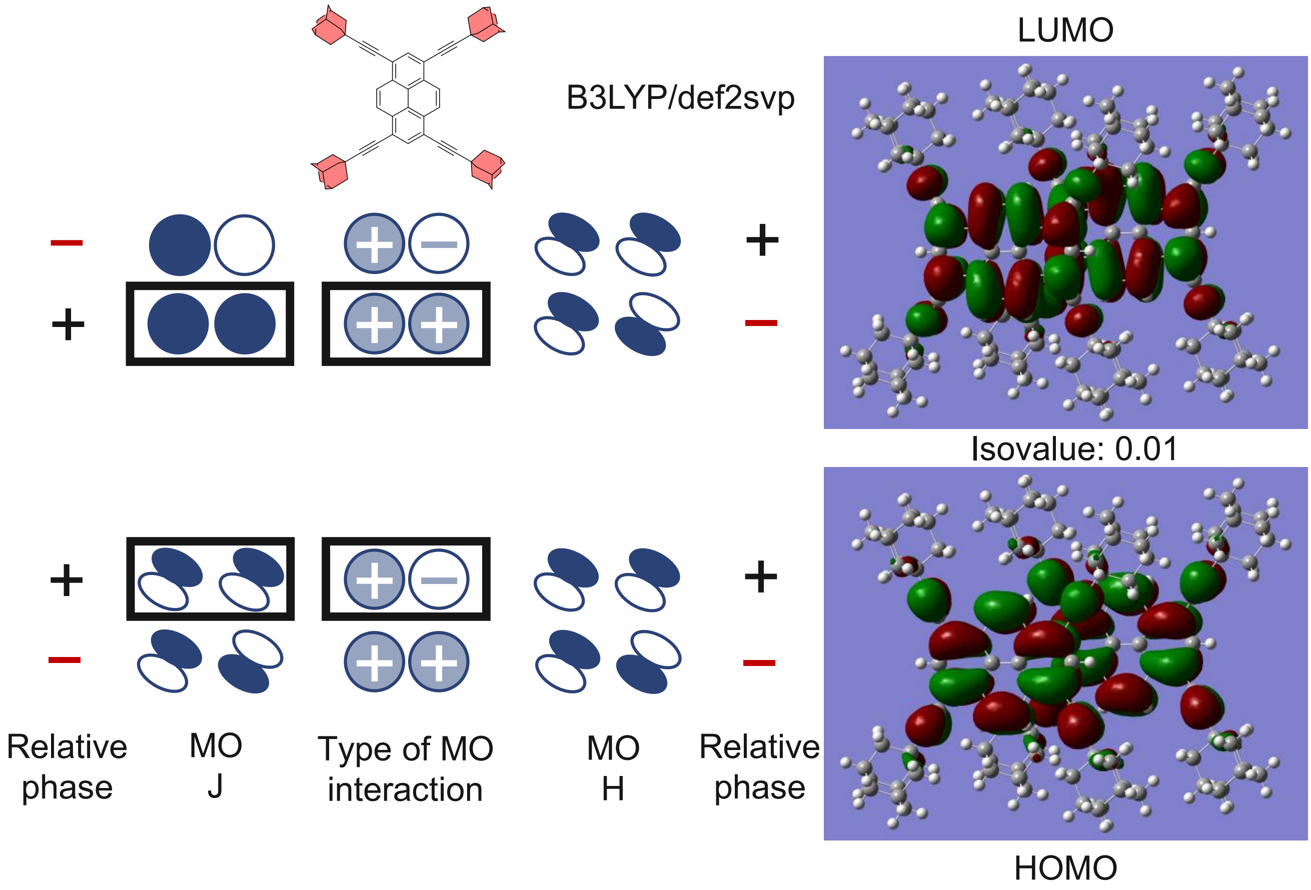

Figure S37 Schematic illustration of the relationship between relative molecular orbital (MO) phase and H-/J-type electronic coupling, together with representative frontier orbital plots for tetrakis(adamantylethynyl)-substituted pyrene **4**. Left: Conceptual depiction of the relative phase of neighboring frontier orbitals and the resulting type of MO interaction. The overall relative phase of the two monomers in a stacked dimer depends on the local orbital interactions and is out-of-phase for HOMO–HOMO interactions (destabilization, higher energy level) and in-phase for LUMO–LUMO interactions (stabilization, lower energy level). For **4**, the overall phase relationship of the two monomers is preserved, as indicated by the black boxes, corresponding to J-type charge-transfer coupling ($t_{\mathrm{h}} > 0$, $t_{\mathrm{e}} > 0$). Right: Calculated LUMO (top) and HOMO (bottom) isosurfaces for a representative dimer at the B3LYP/def2svp level of theory (isovalue = 0.01). The consistent phase relationship between neighboring molecules for both frontier orbitals results in transfer integrals of the same sign for electrons and holes (t_{e} and t_{h}).

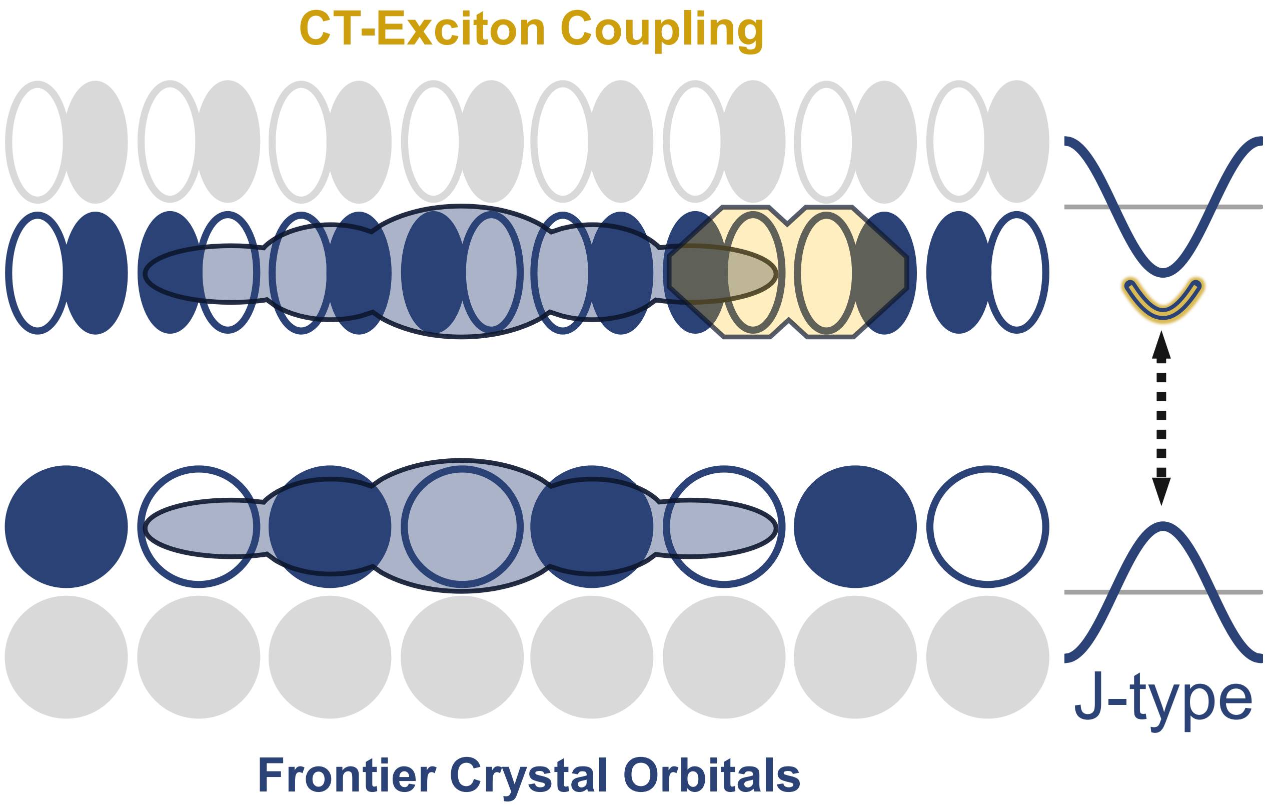

Figure S38. Schematic illustration of J-type band formation and exciton coupling in a one-dimensional molecular stack. The upper part depicts the conduction band derived from LUMO orbitals with p-type symmetry, while the lower part depicts the valence band derived from HOMO orbitals with s-type symmetry. The formation of frontier crystal orbitals delocalized over approximately five molecules is illustrated as semi-transparently filled blue clouds for both the conduction and valence bands, representing band-like orbital delocalization along the stacking direction. Preservation of the overall molecular orbital phase for both HOMOs and LUMOs along the stack leads to in-phase local orbital interactions in the conduction band, resulting in energetic stabilization, and out-of-phase interactions in the valence band, resulting in energetic destabilization. Exciton coupling between neighboring molecules, encompassing both Coulombic and charge-transfer–mediated interactions, is illustrated by semi-transparently filled yellow regions at intermolecular contacts. The band diagram on the right schematically summarizes the resulting energy lowering arising from the combined effects of band formation and exciton coupling, yielding a reduced band gap characteristic of J-type electronic behavior.

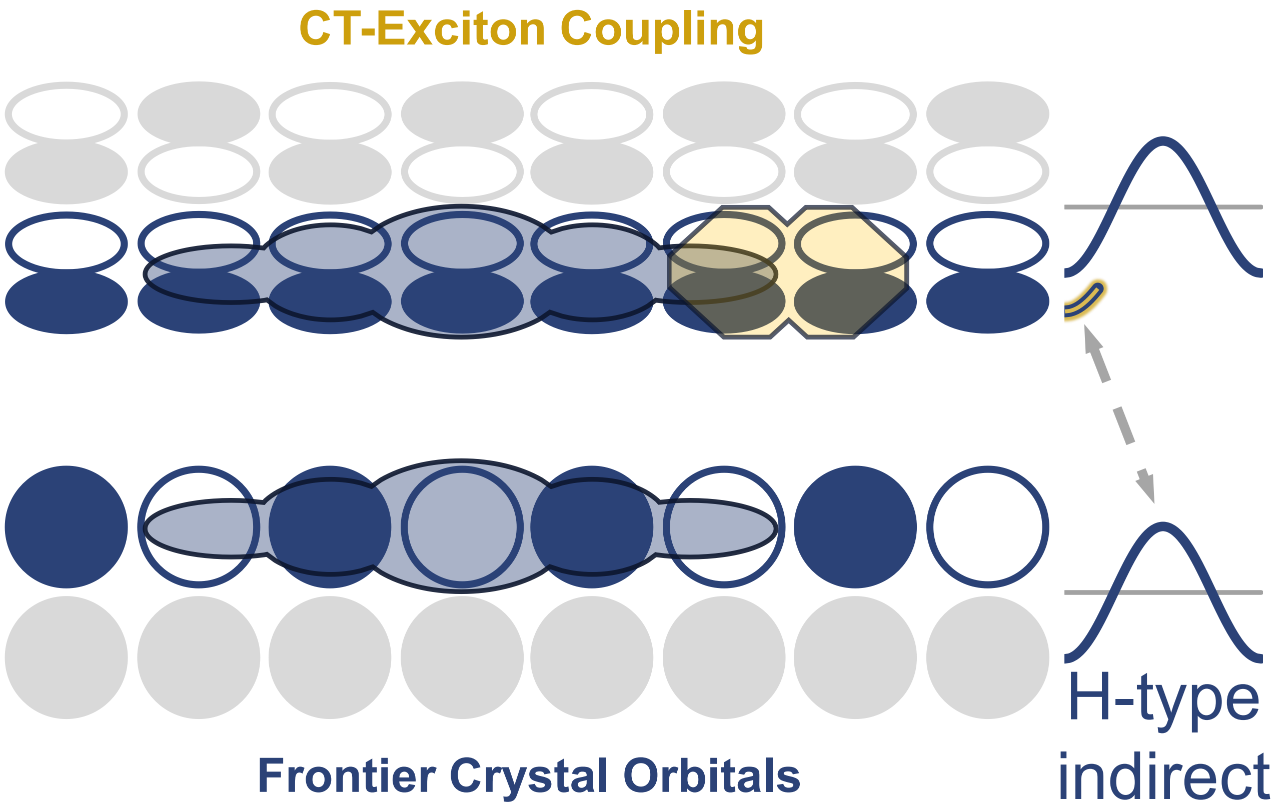

Figure S39. Schematic illustration of H-type band formation and exciton coupling in a one-dimensional molecular stack, leading to an indirect lowest-energy transition. The upper part depicts the conduction band derived from LUMO orbitals with p-type symmetry, while the lower part depicts the valence band derived from HOMO orbitals with s-type symmetry. The formation of frontier crystal orbitals delocalized over approximately five molecules is illustrated as semi-transparently filled blue clouds for both the conduction and valence bands, representing band-like orbital delocalization along the stacking direction. In contrast to the J-type case, in-phase local interactions of LUMOs lead to an overall phase inversion between HOMOs and LUMOs along the molecular stack, resulting in H-type electronic coupling. Exciton coupling between neighboring molecules, encompassing both Coulombic and charge-transfer–mediated interactions, is illustrated by semi-transparently filled yellow regions at intermolecular contacts. The band diagram on the right schematically summarizes the resulting electronic structure, in which the band extrema occur at different crystal momenta, giving rise to an H-type, indirect lowest-energy transition, despite an overall narrowing of the electronic gap due to band formation and excitonic effects.

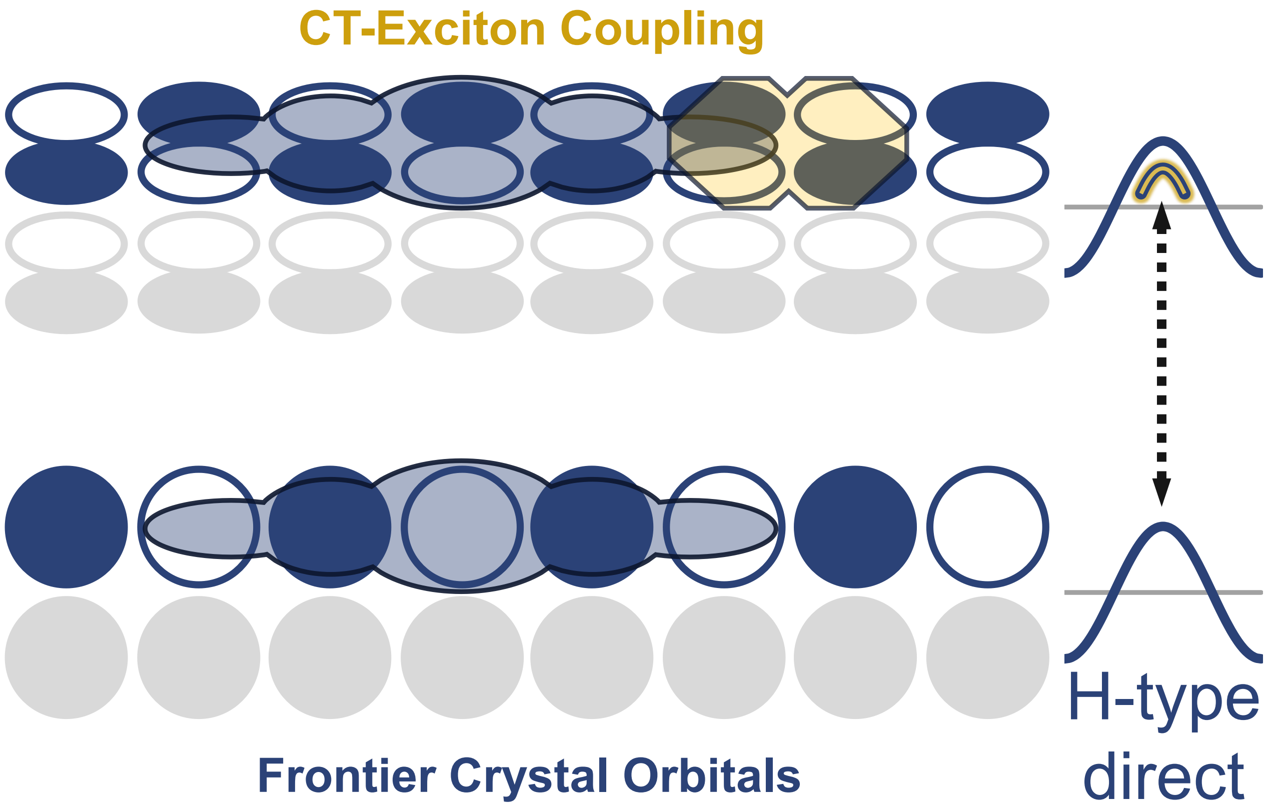

Figure S40. Schematic illustration of H-type band formation and exciton coupling in a one-dimensional molecular stack leading to a *direct* lowest-energy transition. The upper part depicts the conduction band derived from LUMO orbitals with p-type symmetry, while the lower part depicts the valence band derived from HOMO orbitals with s-type symmetry. The formation of frontier crystal orbitals delocalized over approximately five molecules is illustrated as semi-transparently filled blue clouds for both the conduction and valence bands, representing band-like orbital delocalization along the stacking direction. In this case, the overall molecular orbital phase arrangement results in coincident band extrema at the same crystal momentum, yielding an H-type band topology with a direct lowest-energy transition, despite the non-J-type phase relationship between HOMOs and LUMOs. Exciton coupling between neighboring molecules, encompassing both Coulombic and charge-transfer–mediated interactions, is illustrated by semi-transparently filled yellow regions at intermolecular contacts. The band diagram on the right schematically summarizes the resulting electronic structure, showing a direct transition at the band edge accompanied by an overall narrowing of the electronic gap due to the combined effects of band formation and exciton coupling.

Table S7. Calculated frontier orbital energies and HOMO–LUMO gaps of the representative molecule under different dielectric environments.

	ε_{opt}	HOMO	LUMO	E_{gap}
DCM	2.03	−5.350	−2.362	2.988
Solid	3.00	−5.236	−2.244	2.993
Vacuum	1.00	−5.034	−2.036	2.998

[a] ε_{opt}: Optical dielectric constant used to model the environment, corresponding to dichloromethane (DCM), crystalline solid, and vacuum, respectively. HOMO and LUMO: Energies of the highest occupied and lowest unoccupied molecular orbitals calculated by DFT at the B3LYP/def2svp level of theory using the same molecular geometry. E_{gap}: HOMO–LUMO gap defined as $E_{\mathrm{LUMO}} - E_{\mathrm{HOMO}}$. All energies are given in eV. The solid-state dielectric constant represents an effective optical dielectric screening used to approximate the crystal environment.

Author contributions

Benedikt Herbert: formal analysis (synthesis, spectroscopy, crystallography); investigation (synthesis, spectroscopy, crystallography); visualization; writing – original draft preparation; writing – review & editing. **Kazutaka Shoyama**: conceptualization; formal analysis (spectroscopy, crystallography, quantum chemical calculations); funding acquisition; investigation (spectroscopy, crystallography, quantum chemical calculations); supervision; visualization; writing – original draft preparation; writing – review & editing.

References

S1 J. P. Schaefer, M. J. Dagani, D. S. Weinberg, "Secondary isotope effects in the solvolysis of norbornyl bromides" *J. Am. Chem. Soc.* **1967**, *89*, 6938–6944.

S2 W. A. Chalifoux, M. J. Ferguson, R. McDonald, F. Melin, L. Echegoyen, R. R. Tykwinski, "Adamantyl-endcapped polyynes" *J. Phys. Org. Chem.* **2012**, *25*, 69–76.

S3 K. Thakur, D. Wang, S. Mirzaei, R. Rathore, "Electron-Transfer-Induced Self-Assembly of a Molecular Tweezer Platform" *Chem. A Eur. J.* **2020**, *26*, 14085–14089.

S4 W. Kabsch, "XDS" *Acta Crystallogr. D* **2010**, *66*, 125–132.

S5 G. M. Sheldrick, "SHELXT – Integrated space-group and crystal-structure determination" *Acta Crystallogr. A* **2015**, *71*, 3–8.

S6 G. M. Sheldrick, "Crystal structure refinement with SHELXL" *Acta Crystallogr. C* **2015**, *71*, 3–8.

S7 C. B. Hübschle, G. M. Sheldrick, B. Dittrich, "ShelXle: a Qt graphical user interface for SHELXL" *J. Appl. Crystallogr.* **2011**, *44*, 1281–1284.

S8 A. L. Spek, "checkCIF validation ALERTS: what they mean and how to respond" *Acta Crystallogr. E* **2020**, *76*, 1–11.

S9 A. L. Spek, "Single-crystal structure validation with the program PLATON" *J. Appl. Crystallogr.* **2003**, *36*, 7–13.

S10 Gaussian 16, Revision A.03, M. J. Frisch, G. W. Trucks, H. B. Schlegel, G. E. Scuseria, M. A. Robb, J. R. Cheeseman, G. Scalmani, V. Barone, G. A. Petersson, H. Nakatsuji, X. Li, M. Caricato, A. V. Marenich, J. Bloino, B. G. Janesko, R. Gomperts, B. Mennucci, H. P. Hratchian, J. V. Ortiz, A. F. Izmaylov, J. L. Sonnenberg, D. Williams-Young, F. Ding, F. Lipparini, F. Egidi, J. Goings, B. Peng, A. Petrone, T. Henderson, D. Ranasinghe, V. G. Zakrzewski, J. Gao, N. Rega, G. Zheng, W. Liang, M. Hada, M. Ehara, K. Toyota, R. Fukuda, J. Hasegawa, M. Ishida, T. Nakajima, Y. Honda, O. Kitao, H. Nakai, T. Vreven, K. Throssell, J. A. Montgomery, Jr., J. E. Peralta, F. Ogliaro, M. J. Bearpark, J. J. Heyd, E. N. Brothers, K. N. Kudin, V. N. Staroverov, T. A. Keith, R. Kobayashi, J. Normand, K. Raghavachari, A. P. Rendell, J. C. Burant, S. S. Iyengar, J. Tomasi, M. Cossi, J. M. Millam, M. Klene, C. Adamo, R. Cammi, J. W. Ochterski, R. L. Martin, K. Morokuma, O. Farkas, J. B. Foresman, and D. J. Fox, Gaussian, Inc., Wallingford CT, 2016.

S11 Y. Shao, Z. Gan, E. Epifanovsky, A. T. B. Gilbert, M. Wormit, J. Kussmann, A. W. Lange, A. Behn, J. Deng, X. Feng, D. Ghosh, M. Goldey, P. R. Horn, L. D. Jacobson, I. Kaliman, R. Z. Khaliullin, T. Kuś, A. Landau, J. Liu, E. I. Proynov, Y. M. Rhee, R. M. Richard, M. A. Rohrdanz, R. P. Steele, E. J. Sundstrom, H. L. Woodcock, P. M. Zimmerman, D. Zuev, B. Albrecht, E. Alguire, B. Austin, G. J. O. Beran, Y. A. Bernard, E. Berquist, K. Brandhorst, K. B. Bravaya, S. T. Brown, D. Casanova, C.-M. Chang, Y. Chen, S. H. Chien, K. D. Closser, D. L. Crittenden, M. Diedenhofen, R. A. DiStasio, H. Do, A. D. Dutoi, R. G. Edgar, S. Fatehi, L. Fusti-Molnar, A. Ghysels, A. Golubeva-Zadorozhnaya, J. Gomes, M. W. D. Hanson-Heine, P. H. P. Harbach, A. W. Hauser, E. G. Hohenstein, Z. C. Holden, T.-C. Jagau, H. Ji, B. Kaduk, K. Khistyaev, J. Kim, J. Kim, R. A. King, P. Klunzinger, D. Kosenkov, T. Kowalczyk, C. M. Krauter, K. U. Lao, A. D. Laurent, K. V. Lawler, S. V. Levchenko, C. Y. Lin, F. Liu, E. Livshits, R. C. Lochan, A. Luenser, P. Manohar, S. F. Manzer, S.-P. Mao, N. Mardirossian, A. V. Marenich, S. A. Maurer, N. J. Mayhall, E. Neuscamman, C. M. Oana, R. Olivares-Amaya, D. P. O'Neill, J. A. Parkhill, T. M. Perrine, R. Peverati, A. Prociuk, D. R. Rehn, E. Rosta, N. J. Russ, S. M. Sharada, S. Sharma, D. W. Small, A. Sodt, T. Stein, D. Stück, Y.-C. Su, A. J. W. Thom, T. Tsuchimochi, V. Vanovschi, L. Vogt, O. Vydrov, T. Wang, M. A. Watson, J. Wenzel, A. White, C. F. Williams, J. Yang, S. Yeganeh, S. R. Yost, Z.-Q. You, I. Y. Zhang, X. Zhang, Y. Zhao, B. R. Brooks, G. K. L. Chan, D. M. Chipman, C. J. Cramer, W. A. Goddard, M. S. Gordon, W. J. Hehre, A. Klamt, H. F. Schaefer, M. W. Schmidt, C. D. Sherrill, D. G. Truhlar, A. Warshel, X. Xu, A. Aspuru-Guzik, R. Baer, A. T. Bell, N. A. Besley, J.-D. Chai, A. Dreuw, B. D. Dunietz, T. R. Furlani, S. R. Gwaltney, C.-P. Hsu, Y. Jung, J. Kong, D. S. Lambrecht, W. Liang, C. Ochsenfeld, V. A. Rassolov, L. V. Slipchenko, J. E. Subotnik, T. V. Voorhis, J. M. Herbert, A. I. Krylov, P. M. W. Gill, M. Head-Gordon, "Advances in molecular quantum chemistry contained in the Q-Chem 4 program package" *Mol. Phys.* **2015**, *113*, 184–215.

S12 E. J. Baerends, N. F. Aguirre, N. D. Austin, J. Autschbach, F. M. Bickelhaupt, R. Bulo, C. Cappelli, A. C. T. van Duin, F. Egidi, C. F. Guerra, A. Förster, M. Franchini, T. P. M. Goumans, T. Heine, M. Hellström, C. R. Jacob, L. Jensen, M. Krykunov, E. van Lenthe, A. Michalak, M. M. Mitoraj, J. Neugebauer, V. P. Nicu, P. Philipsen, H. Ramanantoanina, R. Rüger, G. Schreckenbach, M. Stener, M. Swart, J. M. Thijssen, T. Trnka, L. Visscher, A. Yakovlev, S. van Gisbergen, "The Amsterdam Modeling Suite" *J. Chem. Phys.* **2025**, *162*, 162501.

S13 GaussView, Version 6.0.16, Roy Dennington, Todd A. Keith, and John M. Millam, Semichem Inc., Shawnee Mission, KS, 2016.

S14 N. J. Hestand, F. C. Spano, "Expanded Theory of H- and J-Molecular Aggregates: The Effects of Vibronic Coupling and Intermolecular Charge Transfer" *Chem. Rev.* **2018**, *118*, 7069–7163.

S15 M. E. Madjet, A. Abdurahman, T. Renger, "Intermolecular Coulomb Couplings from Ab Initio Electrostatic Potentials: Application to Optical Transitions of Strongly Coupled Pigments in Photosynthetic Antennae and Reaction Centers" *J. Phys. Chem. B* **2006**, *110*, 17268–17281.

S16 T. Lu, F. Chen, "Multiwfn: A multifunctional wavefunction analyzer" *J. Comput. Chem.* **2012**, *33*, 580–592.
